# Let the Stones Shine: Assessing the Potential of Microwear Analysis on Flint Artifacts to Refine the Post-depositional History of Paleolithic Sites

**DOI:** 10.1007/s41982-025-00225-2

**Published:** 2025-08-26

**Authors:** Mickael Baillet, Areti Leventi, Hong Chen, Marie Soressi

**Affiliations:** 1 School of Art and Archaeology, Zhejiang University, 148 Tianmushan Rd, Hangzhou, 310028 China; 2 Faculty of Archaeology, Leiden University, Einsteinweg 2, 2333CC Leiden, Netherlands; 3 University of Bordeaux, UMR5199 PACEA Pessac, France

**Keywords:** Traceology, Lithic study, Surface alteration, Post-depositional processes, Taphonomy

## Abstract

Microwear analysis of lithic artifacts offers potential to reconstruct the post-depositional history of prehistoric assemblages, as flint’s microcrystalline structure preserves mechanical and chemical traces over time. While micro-use-wear studies have long examined tool function, their application to taphonomic processes remains underexplored. The techniques and protocols used in use-wear studies could be applied to reconstruct the taphonomic life-history of these artifacts, but this approach has not yet become mainstream. Here, we perform an in-depth review of use-wear literature and report our findings with organizing them into four main issues: the debates on the mechanical versus chemical origin of post-depositional polishes; the difficulties with classification or quantification of surface and ridges alterations; the distinction of dirt layers from alterations; and the experimental references available for assessing micro-alterations. The limitations we identify allow us to suggest the necessity of performing mineralogical analysis of post-depositional polishes to confirm their origin, systematic high-magnification assessment of alterations on surfaces/ridges—for instance using a simple ordinal scale to facilitate the analysis of large samples of artifacts—and adopting an efficient cleaning protocols prior to microwear analysis. We also conclude that there is a need to design and perform in vitro replication of natural polishes under controlled conditions that mimic the conditions of prehistoric sites in low entropy system close to “primary contexts.” With this paper, we show that integrating the techniques of microwear analysis into the toolkit of geoarchaeology has the potential to advance the reconstruction of site formation processes.

**Supplementary Information:**

The online version contains supplementary material available at 10.1007/s41982-025-00225-2.

## Introduction

Micro-use-wear analysts have long recognized flint’s exceptional ability to record micro-surface alterations, particularly polish, but also micro-striations and craters, due to flint’s homogeneous microcrystalline structure and low porosity (Keeley, 1980; Marreiros et al., 2015; Trauth et al., 1978). Over the past 50 years, high-power traceological studies have demonstrated that even minimal repetitive use (e.g., cutting meat) generates diagnostic micro-polishes. They have successfully reproduced all types of microwear patterns through extensive experiments. Additionally, minerological analysis confirmed that abrasion is the primary mechanism behind use-wear polish (Masson et al., 1981; Yamada & Sawada, 1993; Schmidt et al., 2020). While use-related micropolishes (e.g., from scraping bone, scraping hide, and cutting plants) are studied in depth (Keeley, 1980; Marreiros et al., 2015), post-depositional micropolishes remain inconsistently defined and underutilized in site formation studies.

The study of macroscopic modifications of artifacts, like fragmentation, edge damage, and the degree of ridge rounding, has long provided insights into the post-depositional history of lithic assemblages, but unfortunately these were performed with the naked eye or at low magnification using a binocular microscope (up to 20× magnification), without utilizing higher magnifications (e.g. Warren, 1905; Pei, 1936; Bordes, 1950; Vignard & Vacher, 1964; Davis, 1967; Borden, 1971; Singer et al., 1973; Clark & Kleindienst, 1974; Hiscock, 1985; Akoshima, 1987; Stein, 1987; Harding et al., 1987; Copeland, 1989; Goren-Inbar et al., 1992; Petraglia & Potts, 1994; Shea, 1999; Villa & Soressi, 2000; Santonja & Pérez-González, 2001; Borrazzo, 2006; Schoville, 2010; Ugalde et al., 2015; Stephan and Naumenko, (Stepanchuk, and Naumenko, 2024); Rose et al., 2025). While macroscopic edge damage and ridge rounding can signal alteration—particularly severe alteration—these features cannot be used to distinguish between causative agents, as similar patterns may result from natural processes such as aeolian or water action (Stapert, 1976; Lenoble, 2005; Chu et al., 2015; Galland et al., 2024), or subsoil movement (Eren et al., 2011; Vallin et al., 2001; Warren, 1905) and human activities like handling (Rots, 2010; Semenov, 1964) or trampling (Tringham et al., 1974; Shea & Klenck, 1993; McPherron et al., 2014). Within these constraints, traceological analysis at medium–low magnifications (75×–145×) allows for a preliminary interpretation of lithic assemblages in “high entropy” conditions (sensu Asher, 1968; Schiffer, 1987; Bertran et al., 2019); this refers to sites in “secondary deposition” that have undergone significant post-depositional reworking—such as high-energy fluvial environments or high-velocity flows in aeolian settings (e.g., Bustos-Pérez & Ollé, 2024; Bustos-Pérez et al., 2019; Chambers, 2005; Chu et al., 2015; Hosfield & Chambers, 2005; Petraglia & Potts, 1994; Shackley, 1974; Stapert, 1976; Venditti et al., 2016; Werner, 2018). Conversely, low-power traceology remains inadequate for “low-entropy” contexts; meaning sites close to their “primary deposition”, shaped by slow-acting, long-term energy systems, often extending over thousands of years (Asher, 1968; Schiffer, 1987; Bertran et al., 2019).

This paper argues that (a) post-depositional micro-alterations on flint artifacts represent an underexploited resource for studying site formation processes and (b) use-wear analytical techniques at high magnification have important potential to nuance and improve the reconstruction of the post-depositional history of artifacts. Our claim is supported by an extensive review of the available literature with a focus on four key issues that need to be overcome:

1. What are the formation mechanisms of post-depositional polishes, and why has this debate impeded traceology’s application in taphonomy? While some researchers attribute soil polish to mechanical abrasion (e.g., Caspar et al., 2003, 2009; Levi-Sala, 1986a, 1986b; Masson, 1981; Michel et al., 2019; Moss, 1983; Vaughan, 1981), others emphasize chemical interactions (e.g., Engel & Sharp, 1958; Semenov, 1964; Hooke et al., 1969; Rottländer, 1975; Aubry et al., 1975; Stapert, 1976; Potter & Rossman, 1977; Perry & Adams, 1978; Goffer, 1980; Meeks et al., 1982; Mansur, 1986; Baeseman, 1986; Bradley & Clayton, 1987; Thiry et al., 2014). This dichotomy, further complicated by unresolved relationships between polishes and white patina, has hindered standardized terminology and typologies. Our review of both hypotheses suggests that post-depositional polishes should often be of mechanical origins and that mineralogical analysis needs to be used more often to confirm this.
2. How can the intensity of post-depositional micro-surface alterations be quantitatively assessed, and why did this persistent challenge hindered traceology’s integration with taphonomy? While some studies have classified micro-alteration intensity using ordinal scales (Shackley, 1974; Bradley et al., 1993; Vallin et al., 2001; Chu et al., 2015; Bustos-Pérez et al., 2019; Baillet et al., 2025; Bachellerie et al., submitted) or attempted to quantify these alterations through micro-surface roughness measurement (Galland et al., 2019; Sellier & Stephant, 2017; White et al., 1998), most microwear studies lack alteration intensity classification methods. We evaluate these approaches, highlight the need for classification schemes that are easy to apply to large samples of artifacts, and propose an efficient classification method.
3. How do the artifacts need to be clean prior to the analysis? Effective cleaning protocols for lithic artifacts prior to microwear analysis remain a persistent methodological challenge in traceology. While artifacts may exhibit sediment coatings, clay-cladding, or calcite layers (Asryan et al., 2017; Masson, 1981; Michel et al., 2019; Plisson, 1985), current cleaning methods vary significantly based on preservation state, coating adherence, and research goals. Our review of several case studies reveals that inconsistent cleaning procedures can impair visual assessments of post-depositional alterations and potentially the subsequent micro-surface roughness measurement. We suggest striking a balance between the current context-sensitive approach and a more precautionary one by first testing intensive cleaning methods on unused waste debitage flakes before adjusting the cleaning protocol for the entire sample.
4. How useful are existing experimental references for assessing post-depositional micro-surface alterations? What methodological improvements are needed to overcome these limitations? Tumbling machines have been systematically used for assessing mechanical abrasion (Shackley, 1974; Anderson-Gerfaud, 1981; Vaughan, 1981; Mansur, 1982, 1986; Plisson, 1985; Schick, 1986; Levi-Sala, 1986a; Plisson & Mauger, 1988; Petraglia & Potts, 1994; Burroni et al., 2002; Donahue & Burroni, 2004; Mazzucco et al., 2013; Venditti et al., 2016; Werner, 2018; Bustos-Pérez et al., 2019; Galland et al., 2024). While these effectively simulate high-energy fluvial environments (Schick, 1986), few experiments have replicated post-depositional processes in low-entropy (primary deposition) contexts. To address this gap, we systematically review experimental approaches (in vivo and in vitro) used for observing flint natural alterations, with particular attention to distinguishing between high- and low-entropy depositional environments.

To summarize, our review mainly highlights the limitations associated with each of these four issues, while we will discuss potential solutions to address them. Finally, we also suggest ways to effectively incorporate taphonomic microwear analysis into artifact studies prior to conduct functional analysis.

## Method

We selected a comprehensive body of literature relevant to the four issues listed above. First, we align with Keeley's (1980) statement that low magnifications are unsuitable for polish examination due to resolution deterioration and poor light intensity, regardless of the incident light angle. We also concur with Borel et al. (2014) that 100× magnification represents the upper limit of low magnifications under reflected light microscopy, whereas high magnification begins at 200× (Borel et al., 2014, p.50). We hence determined 200× magnification as the minimum threshold for identifying and characterizing post-depositional micro-polishes: their texture, coalescence, light reflectivity, directionality, and other micro-features associated. We determined 100× magnification as the upper limit of medium-low magnification ranges, finding it particularly suitable for documenting polish distribution across micro-surfaces. We hence excluded studies relying solely on low-power microscopy (e.g., Warren, 1905; Pei, 1936; Bordes, 1950; Vignard & Vacher, 1964; Davis, 1967; Borden, 1971; Singer et al., 1973; Clark & Kleindienst, 1974; Hiscock, 1985; Akoshima, 1987; Stein, 1987; Harding et al., 1987; Copeland, 1989; Goren-Inbar et al., 1992; Petraglia & Potts, 1994; Shea, 1999; Villa & Soressi, 2000; Santonja & Pérez-González, 2001; Borrazzo, 2006; Schoville, 2010; Ugalde et al., 2015; Stephan & Naumenko, 2024; Rose et al., 2025). These studies document macroscopic wear features (e.g., edge scarring, ridge or edge rounding, macro-cracks, and macro-striations), whereas our review focuses on micro-alterations requiring higher magnification, particularly the polishes.

Second, given the extensive literature, we prioritized relevant sources over exhaustive coverage. We selected key case studies from microwear analysis literature aligning with our research objectives with emphasizing foundational works (1950s–1990s) and post-2000s research on flint artifact post-depositional modification (e.g., visual, chemical, and roughness analysis).

Third, we focused on the most common Eurasian Paleolithic contexts—periglacial, temperate interglacial, colluvial, fluvial, and karstic environments, where “soil sheen polishes”—potentially encompassing a wide range of different post-depositional polishes such as “aeolian abrasion polish” and “water runoff polish”—and white patinas dominate post-depositional modifications. Desert and lacustrine settings, along with colored patinas (attributed to chemical weathering from soil/airborne elements), were then excluded (Engel & Sharp, 1958; Hooke et al., 1969; Perry & Adams, 1978; Potter & Rossman, 1977; Rottländer, 1975; Stapert, 1976).

## Results

### The Controversy over the Mechanical or Chemical Nature of Post-depositional Polishes

The advent of micro-traceology and chemical micro-analysis in the mid-twentieth century (using optical microscopy and SEM for visual analysis, plus spectrometry) demonstrated that many lithic surface modifications were geogenic (Engel & Sharp, 1958; Hooke et al., 1969; Perry & Adams, 1978; Potter & Rossman, 1977; Rottländer, 1975; Semenov, 1964), while earlier attempts were mostly empirical (e.g. Hue, 1929; Meillet, 1866). However, a persistent debate has divided researchers for decades regarding whether certain post-depositional micro-polishes (termed “sheen,” “gloss,” etc., see Table 1) result from mechanical or chemical processes. This controversy remains unresolved for Pleistocene and Early Holocene lithic assemblages.

**Table 1:** Comparison of terminology and formation hypotheses for post-depositional polishes (as observed at high magnification under different microscopes) on flint artifacts

	Magnification	Microscope	Terminology used	Hypothesis of formation	Reference authors
Post-depositional polishes	100× to 500×	Optical	Soil sheen	Mechanical abrasion	Moss (1983); Vaughan (1981); Levi-Sala (1986a, 1986b)
	100× to 5000×	Optical + SEM	Soil sheen	Chemical or mechanical action	Anderson-Gerfaud (1981); Mansur (1986)
	100× to 5000×	Optical + SEM	Surface sheen	Chemical or mechanical action	Plisson (1985); Plisson and Mauger (1988)
	100× to 500×	Optical	Gloss/shiny surface polish/lustre	Chemical or mechanical action	Semenov (1964)
	100× to 500×	Optical	Patination	Chemical or mechanical action	Keeley (1980)
	1000× to 5000×	SEM	Natural gloss/glaze	Chemical action	Meeks et al. (1982)
	100× to 500×	Optical	Soil sheen/veil	Mechanical abrasion	Michel et al. (2019)
	100× to 500×	Optical	Surface polish	Mechanical abrasion	Vallin et al. (2001); Caspar et al. (2003)
	100× to 500×	Optical	Sheen surface/polished surface/glossy surface	Chemical or mechanical action	Burroni et al. (2002)
	100× to 500×	Optical	Glossy patina	Chemical action	Bradley et al. (1993)
	100× to 500×	Optical	Glossy patina/soil sheen	Gloss = chemical action; sheen = mechanical action	Moncel et al. (2023)
	Undetermined	Undetermined	Solution gloss	Chemical action	Shepherd (1972)
	145×	Dino-Lite	Abrasive smoothing	Mechanical abrasion	Bustos-Pérez et al. (2019); Bustos-Pérez and Ollé (2023)
	100× to 500×	Optical	Lustre	Mechanical abrasion	Mazzucco et al. (2013)
	100× to 5000×	Optical + SEM	Lustré de sol	Mechanical abrasion	Masson et al. (1981)
	100× to 5000×	Optical + SEM	Gloss patina	Chemical action	Stapert (1976)
	1000× to 5000×	SEM	Glaçage siliceux	Chemical action	Aubry et al. (1975)
	1000× to 5000×	SEM	Glossy patina	Chemical action	Rottländer (1975)
	1000×	SEM	Surface sheen polish	Mechanical abrasion	Chu et al. (2015)
	1000× to 2000×	SEM	Secondary silica film	Chemical action	Thiry et al. (2014)
	100× to 500×	Confocal	Lustred surface	Chemical or mechanical action	Galland et al. (2019)

Several studies have interpreted post-depositional polishes through microscopy alone, often without experimental validation, but only a citation of the bibliographic source (generally Rottländer, 1975; and/or Levi-Sala, 1986a): Kaminska et al. (1993) attributed polish to periglacial mechanical action; Moncel et al. (2023) distinguished between chemically-formed “glossy patina” and mechanical “soil sheen” without clear visual differentiation (see fig.11-d in Moncel et al., 2023, p. 19); Lhomme et al. (2010) proposed conflicting water flow and cryoturbation interpretations for the same assemblage, depending on the observational scale of alteration features (i.e. 20× vs. 200×) (Lhomme et al., 2010, fig.4d and 4e, p.59); and Stapert (1976) proposed conflicting aeolian abrasion and chemical action interpretations for the same assemblage exhibiting edge and ridge rounding (Stapert, 1976, fig.4, p.15; this article, Supplementary material 1, fig. S2b). These cases highlight persistent methodological challenges in distinguishing mechanical vs. chemical origins of surface modifications through visual analysis alone.

To address this, it is useful to trace the chronological development of theoretical approaches—from the 1950s to the present—grounded in experimental references, with particular emphasis on the contrast between chemical and mechanical theories.

#### The Chemical Theory

Post-depositional polishes are often mischaracterized as “transparent” overlays; in reality, they integrate with the artifact’s microtopography. It has frequently been acknowledged that these micro-surface alterations exhibit physical characteristics consistent with polish formation (i.e., a surface rendered more homogeneous and shiny through mechanical action), as they demonstrate polish-like qualities when observed in plan-view under both metallurgical microscopy and SEM (Keeley, 1980; Rottländer, 1975; Semenov, 1964). Early analysts (1950s–1990s) frequently attributed them to chemical processes, terming them “patinas” or “natural gloss” rather than mechanical polishes (Table 1). Unlike post-depositional polishes, white patina often appears as a transparent “veil” under microscopy, obscuring surface observation (Keeley, 1980; Plisson & Mauger, 1988; Semenov, 1964). While traditionally considered distinct from post-depositional polishes (Hue, 1929; Meillet, 1866; Semenov, 1964), studies since the 1970s increasingly suggest these phenomena may represent related processes along a continuum.

Methodologically, post-depositional polishes and white patinas were analyzed in plan-view or in cross-section via optical microscopy (e.g., Bradley & Clayton, 1987; Mansur, 1986; Semenov, 1964; Stapert, 1976) or SEM (e.g., Rottländer, 1975); Aubry et al., 1975; Potter & Rossman, 1977; Perry & Adams, 1978; Goffer, 1980; Meeks et al., 1982; Mansur, 1986; Baeseman, 1986), while chemical composition was assessed using X-ray diffraction (e.g., Engel & Sharp, 1958; Hooke et al., 1969; Perry & Adams, 1978; Potter & Rossman, 1977). Very few are based on theoretical inferences without practical analysis (Howard, 1999, 2002). Experimental replication of glossy patina, for a duration ranging from three weeks (Rottländer, 1975) to one month (Aubry et al., 1975), proved challenging. Strong acids/bases typically produced white patina, whereas weak solvents (e.g., phenolic acid and deionized water) induced silica dissolution/redeposition without white patina formation (Aubry et al., 1975; Rottländer, 1975). However, these conclusions were based exclusively on SEM imaging (Supplementary Material 1, fig.S1-A). Sieveking and Clayton (1986) conducted freeze-thaw experiments in deionized water without sediment, for 126 days. Based solely on SEM imaging, they reported internal etching but no silica redeposition (Supplementary Material 1, fig.S1-B).

These divergent results prompt a key question: how can similar surface alterations form under varying pH conditions, including neutral water? Rottländer (1975) suggested that some solvents are either too bulky or too weak to infiltrate these microstructures, and that the potential energy gradient between topographic peaks and valleys limits polish formation to a thin “surface layer,” thereby slowing the overall alteration process (Rottländer, 1975, p. 109). In contrast, the formation of white patina, according to Rottländer, occurs when solvents are capable of deep penetration into the microstructure. From this perspective, “gloss patina” and white patina are not mutually exclusive but rather represent different outcomes of similar processes operating under varied physico-chemical conditions. Masson (1981) offered a divergent interpretation based on her SEM and X-ray diffraction analysis of Middle Paleolithic artifacts. Without reference to experimental replication, she found no evidence of silica redeposition. Instead, she identified white patina as the result of silica dissolution, leading to microscale concavities in the surface. Under specific conditions, she further observed post-depositional neogenesis of silica, culminating in the formation of trydimite opal crystals on the order of one micron in size (Masson, 1981, p. 1533).

Plisson (1985) conducted chemical alteration experiments (water flow, phenolic acid, alkaline solutions, etc.) on flint samples over five months. Using optical microscopy (100x–500x) and SEM (5000x), he observed no significant surface alteration or gloss formation. The sole exception was a highly recrystallized Cretaceous chalk flint from the Upper Campanian showing, after the alkaline experience, minor micro-pitting located only in the places where hydrated and highly soluble silica forms occurred (Plisson, 1985, p.130) (Supplementary Material 1, fig.S1-C & S1-D). Replication by Mansur (1986) confirmed these results.

After this first generation of pioneering works, from the beginning of the twenty-first century to the present, the same interrogations have persisted. Mineralogists Thiry et al. (2014), building on the work by Aubry et al. (1975), Masson (1981), and Plisson (1985), but without experimental reference, also highlighted that the various mineralogical components of flint exhibit different degrees of crystallinity and solubility. They emphasized the role of differential silica dissolution, posing that the less stable silica phases are more susceptible to leaching. They framed this process in terms of the principle of Ostwald ripening, whereby the dissolution of weaker silica grains in soil solutions facilitates the redeposition of silica onto stronger grains, promoting their growth (Thiry et al., 2014, p. 147). On the basis of SEM analysis of intentionally cross-sectioned Paleolithic artifacts, they suggested that secondary silica layers (tens of microns thick) develop approximately over 20,000–50,000 years (Supplementary Material 1, fig.S2-A-). However, their chronological estimates rely on comparison with geothermal deposit studies (Herdianita et al., 2000), and on the fact that the critical temperature for solubility of the amorphous silica is 25° (Morey et al., 1964), which are not comparable to the cold to temperate burial contexts of the Eurasian Paleolithic sites, according to Howard (2002). As Howard pointed out: “Silica deposition of such a magnitude would require exposure to water solutions greatly and consistently above normal ambient silica saturation levels without periodic silica content reduction sufficient to trigger desorption. Such a scenario would only occur in the vicinity of silica rich hot springs or geysers, certainly not in the majority of archaeological deposits” (Howard, 2002, p.284). Nevertheless, both Howard’s and Thiry’s theories of post-depositional polish formation align with Rottländer's in proposing silica dissolution-reprecipitation. Other mineralogists, based on in-vitro experiments involving alkaline solution for a duration of 9 days (Caux et al., 2018), or based on archaeological material examined in cross-section (Fiers et al., 2018; Glauberman & Thorson, 2012; Pawlikowski & Wasilewski, 2002), confirmed this mechanism for white patina, but remained inconclusive or silent regarding post-depositional polishes. Microwear analysts Mazzucco et al. (2013) experimentally tested alkali-silica reaction on flint using pine wood ash solution (i.e., enriched with calcium, potassium, sodium, and magnesium) for more than 11 days. On the basis of low-magnification thin-section observations (10×) under a transmitted light microscope, and high-magnification (500x) observations under a metallurgical microscope, they reported dissolution patterns affecting the weaker silica phases, but no polish formation nor micro-pitting. Drawing from Prezzi et al. (1997), who studied the alkali-silica reaction in concrete—a material composed of artificially cemented aggregates—Mazzucco et al. (2013) proposed a “silica gel” reprecipitation model for white patina on flint, though concrete-derived analogies remain speculative. In contrast, Asryan et al. (2017), through a two-year in vitro experiment involving acidic phosphatic corrosion (from guano), and subsequent traceological analysis of the flint artifacts at high-magnifications under metallurgical microscopy (≥200x) and SEM analysis (≥1000x), reported white patina and micropitting—but no polish. These findings were corroborated by archaeological specimens from Azokh Cave (~180–100 ka) (Asryan et al., 2017).

In sum, while several mineralogists or microwear analysts (e.g., Rottländer, 1975; Aubry et al., 1975; Stapert, 1976; Burroni et al., 2002; Mansur, 1986; Thiry et al., 2014) and archaeologists (e.g., Honea, 1964; Shepherd, 1972) proposed that flint “gloss patina” forms through silica amorphization and redeposition, some experimental reproductions and SEM evidence remain contested (Mazzucco et al., 2013; Plisson, 1985). The distinction between post-depositional polishes and white patina remains debated (Caux et al., 2018; Fiers et al., 2018; Glauberman & Thorson, 2012; Mazzucco et al., 2013; Rottländer, 1975; Thiry et al., 2014). Micro-cavities (1–5 µm) appear primarily associated with white patina (Asryan et al., 2017; Keeley, 1980; Mansur, 1986; Masson, 1981; Plisson, 1985), though inconsistently observed (Galland et al., 2019; Plisson & Mauger, 1988).

#### The Mechanical Theory

While Shackley’s (1974) tumbling experiments lacked high-magnification analysis, Shackley first attributed such polishes to abrasion. Masson et al. (1981) later used SEM and X-ray diffraction analysis to distinguish between anthropogenic cereal-harvesting polish and “soil sheen” on Neolithic flint artifacts, finding no silica layer and concluding the latter was purely mechanical—a view recently supported by Schmidt et al.’s (2020) infrared spectroscopy. Though unconfirmed by XRD, Masson et al. (1981) extended this mechanical interpretation to soil sheen formation. Meeks et al. (1982) experimentally reproduced “natural gloss” using in vitro mechanical abrasion (metallurgical polisher impregnated with 6 µm/1 µm diamond paste), and observed the experimental pieces and Natufian artifacts both in plan view and cross-section under the SEM. Moss (1983), Vaughan (1981), and Levi-Sala (1986a, 1986b) attributed similar “soil sheen” to sediment abrasion, supported by tumbling experiments. Subsequent studies (Bustos-Pérez et al., 2019; Chu et al., 2015; Mazzucco et al., 2013) confirmed these findings, also on the basis of in vitro motorized mechanical experiments, and microscopy at medium-low (145x), high magnification (200–500x), or very high magnification (1000x). Freeze-thaw cycle experiments (conducted both in vivo and in vitro for durations ranging from 75 days to 4 years) induced a very light mechanical abrasion on flint artifacts through their displacement in subsoil (silty sediment) (Supplementary Material 1, fig.S3-B). However, some specimens showed no detectable polish at high magnifications (≥200x) under metallurgical microscopy (Baillet et al., 2025; Caspar et al., 2003, 2009; Claud & Bertran, 2010; Michel et al., 2019; Texier et al., 1998; Vallin et al., 2013).

It is noteworthy that micro-craters and micro-striations are post-depositional surface modifications primarily formed by mechanical sediment action (Levi-Sala, 1986a; Mansur, 1982; Plisson & Mauger, 1988). Micro-craters, typically exceeding 10 µm in diameter, are most clearly observable under metallurgical microscopy at high magnifications (≥200x) (e.g. Baillet et al., 2025; Caspar et al., 2003; Levi-Sala, 1986a; Mazzucco et al., 2013; Plisson & Mauger, 1988).

#### Undetermined Positions

Some researchers maintain a neutral stance on polish formation, using non-committal terms like “surface sheen” or “surface alteration” (Burroni et al., 2002; Coffey, 1994; Plisson, 1985). While Rottländer (1975) acknowledged chemical origins, he noted mechanical similarities, possibly referencing Shackley (1974). Interestingly, Burroni et al. (2002) also took an intermediate position between the mechanical and chemical hypothesis, drawing ambiguous parallels between flint surfaces and industrial ceramic wear (Blomberg et al., 1993), despite compositional differences (Masson et al., 1981). Blomberg et al. (1993), on the basis of a controlled experimental approach and SEM analysis of ceramic and glass surfaces, proposed that the mechanical friction of both fine wear debris and tribo-chemically formed compounds causes their sintering and milling, through a dynamic mechanical and chemical process, leading to the formation of a thin film on the surface (called the Beilby layer). This confusion underscores a fundamental principle: weathering processes vary significantly among different rock types (e.g., sedimentary rocks, amorphous silica, and metamorphic rocks) and thus cannot be directly compared, which is particularly the case for example between flint, glass (Suratwala et al., 2015), and hornfels (Gauthier and Burke, 2011). Similar ambiguity appears in Stapert’s (1976) Dutch case study, attributing micro-features to both mechanical polishing and chemical processes.

### Review of Attempts to Systematically Quantify or Classify Micro-surface Alteration Intensity

This section critically reviews methods (1950s–present) for quantifying post-depositional surface alterations in lithics, hypothesizing that microwear analysts adapted at least some use-wear techniques to natural modifications. In functional microwear studies, the matter of relative intensity of use has been addressed since pioneering works through the following: (1) visual assessment at high magnification to evaluate polish coalescence and micro-relief flatness (e.g., Semenov, 1964; Vaughan, 1981), (2) spectroscopic measurement of polish thickness (e.g., Christensen et al., 1992), and (3) quantitative measurement of surface roughness or smoothness (Beyries et al., 1988). However, the hypothesis that use intensity can be inferred from use-wear polish characteristics (i.e., micro-relief flatness, polish thickness, and texture) has been partially disproven (Ibañez & Mazzucco, 2021).

It would seem that the first attempt to systematically classify lithic artifacts according to their degree of alteration at a microscopic scale was made by a non-specialist in microwear analysis (Shackley, 1974). Shackley’s systematic classification of lithic micro-alteration, based on a tumbling experiment, proposed a six-category scale for dorsal ridge rounding, each corresponding to a width interval of the rounding of the dorsal ridge measured with a reticule in the eyepiece of the microscope, at medium-low magnification (75x), as follows: 1 (“very fresh”, “10- 20 µm”); 2 (“fresh”; “20-50 µm”); 3 (“slight abrasion”; “50-100 µm”); 4 (“abraded”; “100-200 µm”); 5 (“heavily abraded”; “200-300”); and 6 (“very heavily abraded”; “300 to >300”) (Shackley, 1974). Shackley successfully applied this “abrasion index” to large archaeological lithic assemblages (*n* > 100 artifacts) in gravel deposit contexts, suggesting that they were stream rolled and re-deposited in secondary context (Shackley, 1974, 1975). Though validated by subsequent tumbling experiments (Anderson-Gerfaud, 1981; Plisson, 1985; Mansur, 1986; Levi-Sala, 1986a), Shackley’s method was cited but not used by pioneering specialists of use-microwear (e.g. Keeley, 1980; Moss, 1983; Vaughan, 1981). While Moss (1983) recognized ubiquitous “soil sheen” as mechanical background noise, she provided no classification method. Paradoxically, Vaughan (1981) developed a use-wear polish intensity index but didn’t apply it to soil sheen, despite describing it as abrasive polish.

Since Shackley (1974), few models have quantified flint micro-surface alteration (e.g., Baillet et al., 2025; Bradley et al., 1993; Burroni et al., 2002; Bustos-Pérez & Ollé, 2024; Bustos-Pérez et al., 2019; Chu et al., 2015; Donahue & Burroni, 2004; Vallin et al., 2001). Donahue and Burroni (2004) and Vallin et al. (2001) proposed classification systems based on experimental abrasion (respectively tumbling and manual abrasion) and microscopic analysis (200×). Donahue’s binary scale (“mild” vs. “severe wear”) resembles Semenov’s (1964) unsystematic approach. Vallin’s four-tier scale (0–3) was inspired by the same scale designed by Bradley et al. (1993) without experimental reference, as follows: category 0 (“resembles closely the freshly fractured surface”); category 1 (“start of the glossy patination and from the slightly smoothed aspects”); category 2 (“lustrous surface which is generally quite uniformly shiny but still preserves a degree of microrelief”); and category 3 (“mirror-smooth surface with some remnants of the original microtopography”) (Bradley et al., 1993). The application of these scales to hundreds of archaeological pieces successfully enabled spatial analysis of differential preservation according to the locus at Tattershall Thorpe site (Bradley et al., 1993), Upper Ninepence site (Donahue & Burroni, 2004), and Champ Bruquette site (Baillet et al., 2025; Vallin et al., 2001).

Despite their methodological advances, Bradley et al. (1993), Vallin et al. (2001), and Donahue and Burroni (2004) failed to visually document polish distinctions in their taphonomic categories, limiting reproducibility. While this limitation might explain the long absence of adoption of their method, their coding systems remain effective and have been recently successfully applied for assessing post-depositional processes across prehistoric sites (Discamps et al., 2019; Bachellerie et al., submitted; Baillet et al., 2025) (Supplementary Material 1, fig.S6).

More recent experimental protocols for classifying flint micro-abrasion (Bustos-Pérez et al., 2019; Chu et al., 2015) show methodological divergences. While both used mechanical devices, they employed different microscopy (SEM at 1000x vs. Dino-Lite at 145x), yielding incompatible traceological attributes. Bustos-Pérez et al. (2019) prioritized dorsal ridge width and edge macroscopic damage over abrasive smoothing (unrelated to micro-polish due to low magnification), whereas Chu et al. (2015) considered surface sheen polish as the primary diagnostic feature. Chu et al. (2015) classify surface polish into four stages (0–3) based on abrasion degree and microtopography leveling as follows: 0 (“no damage,” “no change”); 1 (“slightly abraded,” “less than 33% of the original surface removed”); 2 (“moderately abraded,” “between 33% and 66% of the original surface removed”); and 3 (“highly abraded,” “66% or more of the original surface removed”). However, regardless of the very high magnification used, their SEM-based criteria poorly capture polish texture, coalescence, and shine—features more discernible via metallurgical microscopy (Borel et al., 2014; Ollé et al., 2016). The defined categories of alteration are difficult to differentiate, particularly between those which directly follow one another, and we can only clearly differentiate between extremely opposite categories. Notably, stage 3 polish appears smooth in SEM but may conceal micro-pitting, which is consistently reported in artifacts from tumbling experiments observed under optical microscopy (e.g., Anderson-Gerfaud, 1981); Vaughan, 1981; Mansur, 1982; Plisson, 1985; Levi-Sala, 1986a; Burroni et al., 2002; Donahue & Burroni, 2004). A similar technical problem arose from the attempts made by Bustos-Pérez et al. (2019) to document surface abrasion using a Dino-Lite USB (145x), since the low resolution, poor contrast, and excessive reflected light hindered polish characterization. While later work (Bustos-Pérez & Ollé, 2024) improved image quality via gray-level processing, qualitative analysis remained limited, mostly due to the medium–low magnification.

Dorsal ridge width measurement at low magnification (75x), and subsequent classification into a six-category established scale, has proven effective for assessing reworking in fluvial contexts archaeological sites (Chambers, 2005; Shackley, 1975). Bustos-Pérez et al. (2019) experimentally refined this approach at medium–low magnification (120x) by measuring ridge width through image processing software, an approach that, by the authors’ own admission, failed to adequately resolve fresh or minimally eroded ridges (see Table 15 in Bustos-Pérez et al., 2019). Burroni et al. (2002) and Donahue et al. (2019) developed an even more precise method using 200× magnification and measuring ridge width directly through the micrometric reticle integrated into the eyepiece. Drawing on our own experience as microwear analysts, we concur with Donahue’s conclusion that 200x magnification is the minimum scale necessary for reliably detecting subtle distinctions between unmodified sharp ridges and slightly rounded ones (Donahue et al., 2019, p. 544). Their six-category classification is as follow: “minimally rounded” (<3.0 µm), “mildly rounded” (3.0–5.9 µm), “moderately rounded” (6.0–9.9 µm), “heavily rounded” (10.0–29.9 µm), “extremely rounded” (30.0–89.9 µm), and “rolled” (≥90 µm) (Donahue et al., 2019). This high-resolution classification was applied to a substantial archaeological sample (*n* = 500) from Mesolithic sites in primary contexts, allowing the authors to assess the degree of disturbance at various loci. Burroni et al. (2002) applied a similar method to another large Mesolithic assemblage from a primary context.

Galland et al. (2019) used confocal microscopy to measure micro-surface roughness of post-depositional polishes on archaeological lithic artifacts. Their protocol was designed to address a specific question at a specific site but is relevant here in our search for quantification at high-magnification of artifact surface state. Galland et al. (2019) measured the micro-surface roughness of 13 archaeological artifacts and compared them with a reference set (*n* = 30) of unused flint and geological specimens exhibiting weathered surfaces or white patina. Scatter plots show partial overlapping roughness values among fresh, weathered, and patinated surfaces within both the experimental reference set (Galland et al., 2019, fig. 5, p. 50) and the archaeological sample (fig. 6, p. 51). These results indicate that the roughness measurements currently used are not yet sensitive enough to clearly distinguish between millennia-old surfaces of archaeological artifacts and geological specimens potentially altered over millions of years. Bustos-Pérez and Ollé (2024) quantitatively analyzed micro-surface roughness on experimentally produced artifacts (*n* = 25) that were mechanically abraded for various durations and geological samples with neocortical surfaces. Bustos-Pérez and Ollé (2024) evaluated roughness from the gray level values of micrographs taken at medium magnification (145×). Using this technique, Bustos-Pérez and Ollé (2024) can clearly set aside extreme cases like “fresh” surfaces and “neocortex” surfaces (Bustos-Pérez & Ollé, 2024, fig.8, p.15), but intermediate degree of abrasion corresponding to what is seen in post-depositional polishes from low-energy context is likely not yet possible to differentiate with such a technique.

In sum, quantitative micro-surface roughness measurements or estimation currently lacks discriminative power and needs to be further refined to be successfully used to differentiate between post-depositional polishes, especially the ones produced in low-energy contexts.

### The Issue of Cleaning Procedures Prior to Microscopic Analysis

Post-depositional polishes have been defined as light-reflective flint surface alterations resistant to acids, bases, and solvents (Vaughan, 1981, p. 132)—a definition unchallenged by subsequent experimental studies. In contrast, at least some anthropogenic use-wear polishes are removable via prolonged alkaline exposure without altering the underlying flint surface (Borras, 1990; Coffey, 1994; Knutsson, 1988; Mansur, 1986; Plisson, 1985; Plisson & Mauger, 1988).

In archaeological contexts, flint artifacts may exhibit sediment coatings, clay-cladding, iron oxides, or calcite layers (Semenov, 1964, p. 24; Keeley, 1980; Masson, 1981; Plisson, 1985; Clemente-Conte, 1997); Asryan et al., 2017) (Supplementary Material 1, fig.S4-A & B). More broadly, such layers can occur on experimental artifacts, especially after prolonged sediment contact (e.g., Michel et al., 2019). Chemically, these superficial “dirty layers” can be differentiated by their removability, regardless of organic/inorganic composition. Optical differentiation between these features and some postdepositional polishes can be challenging, as both exhibit light reflectivity under high-magnification metallurgical microscopy.

Cleaning protocols for studying post-depositional polishes on flint artifacts vary considerably across studies, with some omitting this step entirely (Aubry et al., 1975; Caspar et al., 2003, 2009; Masson, 1981; Moncel et al., 2023; Rottländer, 1975; Stapert, 1976; Taylor et al., 2021; Thiry et al., 2014). This variability reflects both differing research objectives and artifact preservation states. While microwear analysts typically employ minimal cleaning to preserve use-related traces, archaeological specimens sometimes require more intensive treatment due to adherent crusts that obscure surface features (Semenov, 1964, p. 24). Keeley (1980) reported on materials from Hoxne and other Paleolithic sites a “shallow mineral deposit that withstands all the above cleaning procedures,” only removable after prolonged immersion (several hours) in hydrochloric acid (HCl) (Keeley, 1980, p. 11). Our microwear analysis of dozens of Eurasian assemblages—spanning the Late Pleistocene to the Holocene—frequently encountered such obstructions. At Les Cottés cave (France), our unpublished microscopic analysis of 746 flint artifacts from various Upper Paleolithic layers revealed a ubiquitous, light-reflective layer of indeterminate origin that mimicked post-depositional polish (Supplementary Material 1, fig.S5-B). Although resistant to initial mild cleaning (brief immersion in hot soapy water followed by brushing under pure water and subsequent alcohol treatment), this layer was effectively removed through short sodium carbonate (Na₂CO₃) immersion (for a few minutes) and gentle brushing with a soft nylon toothbrush for a few seconds (Supplementary Material 1, fig.S5-D).

Most microwear analysts employ chemical treatments early in artifact cleaning, typically using hot demineralized water with detergent (Semenov, 1964; Keeley, 1980; Plisson, 1985; Mansur, 1986; Levi-Sala, 1986a; Plisson & Mauger, 1988; Knutsson, 1988; Bradley et al., 1993; Burroni et al., 2002; Evans & Donahue, 2005; McDonald and Evans, 2014; Chu et al., 2015; Asryan et al., 2017; Caux et al., 2018; Michel et al., 2019; Bustos-Pérez & Ollé, 2024; Galland et al., 2025). Alternatives include spirit or benzine (Semenov, 1964), hydrogen peroxide (30%) (Mazzucco et al., 2013), hydrochloric acid (3–10%), and alkaline solutions such as sodium hydroxide (NaOH, 1 mol/l) (Bradley et al., 1993; Burroni et al., 2002; Evans & Donahue, 2005; Keeley, 1980; Michel et al., 2019; Plisson, 1985), sometimes followed by ultrasonic cleaning (Keeley, 1980; Levi-Sala, 1986a; Bradley et al., 1993; Knutsson, 1988; Evans & Donahue, 2005; Mazzucco et al., 2013; Michel et al., 2019; Bustos-Pérez & Ollé, 2024; Galland et al., 2025). Mechanical brushing with soft nylon is less documented, though common during excavation. Prolonged alkaline treatments can weaken polishes, making them prone to micro-striations from even light dust friction (Plisson, 1985, p. 126; Mansur, 1986). However, gentle brushing for a few seconds on untreated surfaces typically leaves no detectable surface modification nor alteration of use-wear polishes at high magnifications (≥200x) (Plisson, 1985; Mansur, 1986; Levi-Sala, 1988; Bradley, 1993; Evans & Donahue, 2005; Claud, 2008; McDonald and Evans, 2014). After prolonged (2 minutes) wet brushing without sediment, Pedergnana et al. (2020) reported nanoscale roughness changes affecting 7 out of 10 ISO roughness parameters. However, the authors noted that these modifications were not visible in micrographs at the micrometric scale and did not resemble polish, suggesting that the impact on microwear analysis may be negligible (“the surface modifications due to cleaning would simply disappear in the noise,” Pedergnana et al., 2020, p. 274). Whether these alterations stem from abrasion or nylon deposition remains unresolved.

Following Evans et al.’s classification (Evans & Donahue, 2005; McDonald and Evans, 2014), cleaning methods can be categorized as either mild or harsh. The procedures reviewed in this study align with mild cleaning—or even less rigorous approaches (e.g., Caux et al., 2018; Chu et al., 2015). Notably, the harsh cleaning method, which combines chemical treatment (NH₄OH), ultrasonication, and mechanical brushing (Evans & Donahue, 2005), was not adopted. Evans et al. later refined this protocol, increasing NH₄OH to 10% and adding HCl to ensure complete removal of surface particulates, as verified by surface roughness measurements (McDonald and Evans, 2014).

The reliability of published studies on post-depositional polishes may be compromised by residual surface contamination, particularly when cleaning protocols were minimal (Asryan et al., 2017; Chu et al., 2015) or undocumented (Aubry et al., 1975; Caspar et al., 2003, 2009; Rottländer, 1975; Stapert, 1976). Persistent dirt can obscure critical polish characteristics (e.g., coalescence, reflectivity) during high-magnification microscopy (≥ 200x), potentially distorting observations and surface measurements (Evans & Donahue, 2005; McDonald & Evans, 2014). This suggests that some experimental results and archaeological interpretations on post-depositional polishes (e.g., Masson, 1981; Moncel et al., 2023; Stapert, 1976; Taylor et al., 2021; Thiry et al., 2014) may require re-evaluation following more rigorous cleaning.

### Attempts to Experimentally Approach Micro-surface Alteration and the Need to Replicate Low-Energy Environments

Following Boule’s (1905) pioneering work, most mechanical abrasion experiments have employed tumbling machines (30–80 rpm, simulating fluvial conditions) for durations ranging from 12 hours to 4 days (e.g., Shackley, 1974; Anderson-Gerfaud, 1981; Vaughan, 1981; Mansur, 1982, 1986; Plisson, 1985; Schick, 1986; Levi-Sala, 1986a; Plisson & Mauger, 1988; Petraglia & Potts, 1994; Burroni et al., 2002; Donahue & Burroni, 2004; Mazzucco et al., 2013; Venditti et al., 2016; Werner, 2018; Bustos-Pérez et al., 2019; Galland et al., 2025). Visible smoothing and increased shine typically appear within 12 hours, while well-developed polish with >10 µm micro-craters forms after 50–96 hours (Bustos-Pérez et al., 2019; Chu et al., 2015; Levi-Sala, 1986a; Mazzucco et al., 2013; Shackley, 1974) (Supplementary Material 1, fig.S3-A-). Complementarily, fluvial experiments over long durations (7 months to 3 years) demonstrated limited polish development despite substantial artifact transport (≤ 400 m) (Chu & Hosfield, 2020; Hosfield & Chambers, 2005). While such methods effectively represent high-energy contexts (e.g., Shackley, 1975; Schick, 1986; Petraglia & Potts, 1994; Hosfield & Chambers, 2005; Chambers, 2005; Galland et al., 2025), they remain inadequate for low-entropy assemblages. Lenoble’s (2005) overland flow experiments better match periglacial/temperate contexts but lack microwear analysis. Chu et al. (2015) improved upon Schick’s (1986) flume design by fixing artifacts and controlling flow speed, achieving more realistic tribological conditions than tumbling machines, though they exceeded natural flow speeds. However, their experimental model and related classification scale have not been applied to archaeological low-entropy contexts, possibly due to some methodological constraints (see the “Review of Attempts to Systematically Quantify or Classify Micro-surface Alteration Intensity” section).

Sand-blasting experiments (Stapert, 1976; Knutsson & Lindé, 1990; Baillet et al., 2023; Galland et al., 2025) effectively simulate aeolian abrasion in loessic contexts with rapid sedimentation, that is, contexts more or less close to “primary deposition.” Interestingly, microwear analysis conducted on primary deposition sites in the Member F of the Shungura Formation (Ethiopia) (Galland et al. 2025) and Dadong (China) (Baillet et al., 2023), aimed at testing the hypothesis of aeolian abrasion, revealed respectively water runoff and chemical corrosion as dominant taphonomic agents, not wind abrasion. Conversely, studies in the Netherlands and Sweden, where wind is one of the main erosive agents, documented aeolian micro-wear on artifacts (Supplementary Material 1, fig.S2-B). Phillips et al. (2019) demonstrated for African contexts that prolonged wind erosion in sandy environments can displace artifacts over several hundred meters. However, their study did not include high-magnification microwear analysis of the artifacts.

As previously discussed, in vitro experiments of freeze-thaw cycles produce a “gentle alteration sheen polish” on flint artifacts in as few as 50–100 cycles, resembling polish frequently observed on Paleolithic artifacts near primary deposits (Baillet et al., 2025; Caspar et al., 2009; Michel et al., 2019; Vallin et al., 2013). In vivo experiments, however, show no polish due to fewer cycles (Claud & Bertran, 2010; Texier et al., 1998), far below natural cryoturbation cycles (Bertran et al., 2019; Lenoble et al., 2008). Long-term experiments (>1000 cycles) are still needed. In contrast, manual abrasion of flint against frozen sediment or pure frozen water (deionized and distilled) was tested to simulate the mechanical forces and tribological conditions of severe cryoturbation (Baillet et al., 2025; Caspar et al., 2003, 2009). These experiments produced distinct surface modifications: the frozen sediment context generated in only few minutes a mirror-like polish accompanied by fine micro-striations and micro-craters, while the frozen water treatment yielded after 6 hours a gentle polish devoid of striations or craters. Notably, the mirror-like polish closely matches post-depositional alterations observed on archaeological materials from heavily cryoturbated contexts, whereas the “ice polish” closely resembles surface modifications commonly found on Paleolithic artifacts near primary depositional settings (Baillet et al., 2025). Although these experiments provide valuable insights, they do not completely reproduce the frictional conditions characteristic of natural cryoturbation environments.

The “Chemical Theory” section already reviewed experimental approaches examining chemical alteration of flint through exposure to various acidic/alkaline solutions under different durations (Meillet, 1866; Hue, 1929; Engel & Sharp, 1958; Hooke et al., 1969; Shepherd, 1972; Rottländer, 1975; Aubry et al., 1975; Potter & Rossman, 1977; Perry & Adams, 1978; Goffer, 1980; Masson, 1981; Vaughan, 1981; Meeks et al., 1982; Plisson, 1985; Mansur, 1986; Bradley & Clayton, 1987; Plisson & Mauger, 1988; Knutsson, 1988; Borras, 1990; Coffey, 1994; Evans & Donahue, 2005; Mazzucco et al., 2013; Asryan et al., 2017; Caux et al., 2018). Some experimental conditions exceed natural biome variability in agent type/concentration, and the extended timescales of natural processes remain challenging to replicate. Most experiments operate on weekly/monthly timescales, except Hue’s (1929) 20-year study. Laboratory simulations typically compensate the limited timescale through accelerated conditions (increased concentration/temperature) rather than extended durations. Our review indicates that strong acids and bases typically undoubtly produce a white patina. In contrast, weak solvents (e.g., phenolic acid and deionized water) have rarely been reported to induce silica dissolution and redeposition without forming a white patina (Aubry et al., 1975; Rottländer, 1975), though this finding has been challenged (Plisson, 1985). Although purely chemical, frictionless surface alterations have been extensively studied, they remain poorly replicated in experimental settings, and the resulting polishes are often inadequately described, illustrated, and chemically characterized.

## Discussion

The following discussion aims to highlight the trends, gaps, and controversies that we have outlined throughout this article, as well as suggesting some research avenues to overcome the current limitations.

### Toward a Mineralogical Understanding of Post-depositional Polishes

Concerning white patina, while its chemical origin is well-established and successfully replicated experimentally, its precise formation mechanisms remain unclear. The silica redeposition hypothesis (Caux et al., 2018; Fiers et al., 2018; Glauberman & Thorson, 2012; Mazzucco et al., 2013; Thiry et al., 2014) conceptually aligns with earlier theories about post-depositional polish formation (Howard, 1999, 2002; Rottländer, 1975; Stapert, 1976). However, these arguments are limited by (1) reliance on archaeological specimens potentially exhibiting both alteration types without means of discrimination, (2) lack of metallurgical microscope plan-view evidence, and (3) SEM’s inability to distinguish these features at high magnifications (>1000×), where their appearances converge.

Concerning post-depositional polish formation, two main hypotheses exist: chemical and mechanical. The chemical hypothesis, involving silica dissolution/redeposition, micro-pitting, and increased porosity, lacks robust experimental validation. Its support relies primarily on archaeological observations (Glauberman & Thorson, 2012; Howard, 1999, 2002; Stapert, 1976; Thiry et al., 2014) rather than replicated experiments, and visual demonstrations remain inconclusive between microscopy techniques (i.e., metallurgical microscope vs. SEM). Claims of increased porosity, based on visual observations under SEM (Aubry et al., 1975; Thiry et al., 2014), require quantitative verification through methods like Mercury Intrusion Porosimetry (Deprez et al., 2020; Wang & Zhang, 2023) or other advanced techniques (Moses et al., 2014). In contrast, the mechanical hypothesis is well-supported by experimental evidence demonstrating abrasion-induced micropolishes and ridge micro-rounding (Bustos-Pérez & Ollé, 2024; Bustos-Pérez et al., 2019; Caspar et al., 2003, 2009; Chu et al., 2015; Levi-Sala, 1986a; Meeks et al., 1982; Michel et al., 2019; Shackley, 1974).

The origin of post-depositional polishes remains a persistent controversy in traceology. To resolve this, we must determine whether they represent thin amorphous silica deposits or result from abrasive surface alteration without discrete layering. Key questions include the following: (1) If silica-based, are they formed by the deposition of a thin, physically consistent layer—potentially composed of amorphous silica—and if so, what is the thickness of this layer (micrometric vs. angstrom-scale)? (2) If abrasive, are these polishes simply the result of alteration of the surface roughness, enhancing light reflectivity, but without forming any discrete or consistent layer? Schmidt et al. (2020) addressed similar issues for use-wear polishes, finding no evidence of amorphous silica via infrared spectroscopy—a method yet to be applied to post-depositional polishes. This analysis is urgently needed to clarify their formation mechanisms on both experimental and archaeological lithic artifacts.

### Reporting the Degree of Micro-surface Alterations at High-Magnification

**Fig. 1:**
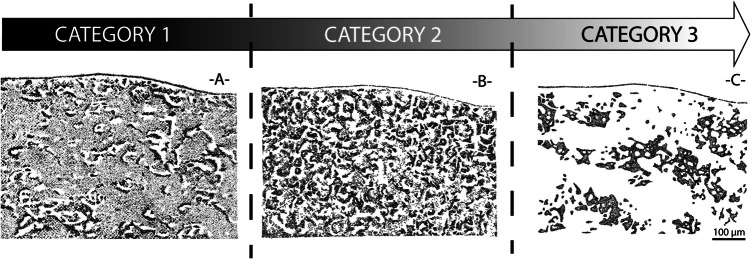
Schematic drawings illustrating three categories of intensity of post-depositional micro-surface abrasion, as observed under a reflected light microscope at high magnification (here shown at 200x). See the text for detailed definitions of each category (Drawing by M.B., inspired by Plisson, 1985, Fig. 1, p. 18)

Quantitative micro-surface roughness measurements (directly from the piece or indirectly from image processing; Bustos-Pérez & Ollé, 2024; Galland et al., 2019) require further technical refinement to conclusively differentiate slightly different micro-surface alterations, like the ones to be expected in low-energy context. While waiting for further development in continuous quantification, we propose to use metallographic microscopy with an ascending ordinal scale of alteration intensity to visually assess the micro-surface abrasion intensity for both surfaces, and ideally ridge-rounding measurements. This could be implemented as a coding index for the degree of micro-surface alteration and/or ridge micro-rounding, facilitating the systematic recording of these data in databases. Concerning ridge width measurement, both the methodology of measurement and the existing coding indices designed by Shackley (1974), Bustos-Pérez et al. (2019), or Donahue et al. (2019) can be applied easily. We have emphasized that the Donahue’s coding index should be prioritized in case of “primary deposition sites,” especially when a very detailed intra-site comparison between artifacts micro-surface abrasion is needed (Baillet et al., 2025; Donahue et al., 2019). Concerning micro-surface abrasion, while the number of categories in classification scales varies depending on site-specific contexts, our own experience demonstrates that a ternary scale often proves both efficient and practical for archaeological applications. These three categories are meant to reflect progressive polish coalescence and intensifying alteration (Fig. 1), as originally designed by Vaughan (1981) and Plisson (1985) for micro-polishes of use:

- Category 1: surface polish is weakly developed and generic, with a loose and diffuse pattern of coalescence on the higher part of the surface topography (Fig. 1A and fig.S6-A). The polish pattern is fluid, reflectivity under incident light is low, and dorsal ridges are generally well preserved (Fig. S6-B).
- Category 2: polish appears moderately developed, forming a “lace-like” coalescence pattern (Fig. 1B and fig.S6-C). Patches moderately coalesce, reflectivity is medium, and dorsal ridges show minor rounding but are still well preserved (Fig. S6-D).
- Category 3: polish is fully developed, with a smooth, homogenous surface texture (Fig. 1C and fig.S6-E). Patches of polish are fully coalescent, and the original surface relief is only retained in the concavities, which may be either original or formed through erosive processes (e.g., macro-quartz detachment or chemical dissolution). Reflectivity is high, and dorsal ridges are significantly rounded (Fig. S6-F).

The integrated method combining the estimation of intensity of post-depositional surface modifications (either micro-surface abrasion or ridge micro-rounding) and its classification into an ascending ordinal scale has been successfully time-tested on large lithic assemblages (*n* > 100 artifacts) from primary-context prehistoric sites, enabling detailed intra-site comparisons of artifact alteration (Baillet et al., 2025; and Supplementary Material 1, fig.S6). To improve interpretability and reproducibility, ordinal classifications should be accompanied by representative high-magnification microphotographs (≥200x) illustrating each alteration level, as exemplified at les Cottés site (Supplementary Material 1, fig.S6). Such a complete framework would bridge the gap between metric data and traditional microwear interpretation. We further recommend that researchers openly share image datasets and analytical workflows as supplementary materials or via open-access repositories (e.g., Galland et al., 2019; Pedergnana et al., 2020; Bustos-Pérez & Ollé, 2024).

### Toward the Generalization of Appropriate Cleaning Prior to Microwear Analysis

The nature and adhesion of surface deposits vary across archaeological contexts, influenced by lithological factors including sediment composition and soil pH. Microwear analysts should tailor study protocols according to assemblage taphonomy, site geoarchaeological context, related research questions, and required traceological resolution. For fluvial or aeolian contexts where surface rounding predominates, medium–low-power approaches focusing on ridge width can be effective with a mild cleaning protocol (Bustos-Pérez & Ollé, 2024; Bustos-Pérez et al., 2019; Chambers, 2005). Primary context materials, however, require high-magnification examination (≥200×) to detect subtle micropolishes potentially obscured by extraneous micro-particulates. In such cases, we recommend testing harsh cleaning protocols (Evans & Donahue, 2005; Macdonald & Evans, 2014) on debitage waste before applying selective cleaning to analysis areas. Chemical treatments should be brief (a few minutes) to avoid compromising post-depositional polishes, followed by short ultrasonic bath. If ultrasonic is not available, it is also efficient to use a gentle nylon brush cleaning, but for a few seconds only, as prolonged mechanical action may induce undetermined nanoscale alterations (Pedergnana et al., 2020).

To summarize, a universal cleaning protocol is impractical, for all the aforementioned factors. We recommend a context-sensitive approach, adjusting techniques based on site conditions and research objectives. In primary contexts—where post-depositional polishes may be subtle—analysts must meticulously clean target surfaces to avoid interference from extraneous particles, which could distort visual or quantitative analyses. As a precaution, harsh cleaning methods should first be tested on non-diagnostic waste debitage flakes. Before cleaning other lithic artifact categories for micro-surface alteration analysis, a preliminary functional assessment using low-power stereomicroscopy should be performed to detect any visible anthropogenic residues.

### Toward More Robust and Refined Methodological Foundations: Expanding Experimental Approaches in Low-Entropy Settings

Our article did not propose a novel experimental design. Instead, our review of experimental methods employed by traceologists to support taphonomic interpretations reveals that while some approaches have proven effective, others require further refinement and development.

Experimental studies of high-energy depositional contexts (e.g., fluvial and desert) have successfully employed motorized setups (tumbling machines and flume systems) combined with medium–low magnification microscopy (75x–145x) to assess surface erosion and post-depositional processes (Shackley, 1974, 1975; Stapert, 1976; Knutsson & Lindé, 1990; Hosfield & Chambers, 2005; Chambers, 2005; Bustos-Pérez et al., 2019; Bustos-Pérez & Ollé, 2024). However, low-entropy contexts near primary deposition present greater challenges, as conventional methods struggle to replicate the extended timescales and subtle processes (e.g., gentle runoff, limited aeolian activity, minor freeze-thaw cycles, and argilliturbation) characteristic of natural burial environments. In vivo experiments have often failed to generate microwear traces of sufficient development or realism (Chu & Hosfield, 2020; Claud & Bertran, 2010; Lenoble, 2005; Texier et al., 1998). While accelerated wear experiments risk unrealistic tribological conditions—thereby producing experimental polishes that diverge qualitatively from their archaeological counterparts—we propose slow-acting systems with precisely controlled variables, building on previous promising works (e.g., Plisson & Mauger, 1988; Knutsson & Lindé, 1990; Vallin et al., 2013; Chu et al., 2015; Asryan et al., 2017; Michel et al., 2019; Baillet et al., 2023, 2025; Galland et al., 2025). Critical gaps remain in reproducing chemical/frictionless alterations (Aubry et al., 1975; Hue, 1929; Rottländer, 1975) and severe cryoturbation processes (Leventi et al., submitted), with insufficient characterization of resulting polishes. We advocate a multimodal approach combining high-magnification reflected light microscopy (≥200x), quantitative micro-surface roughness analysis, and chemical spectroscopy (e.g., FTIR) for more conclusive interpretations.

### Additional Notes on Technical Limitations Inherent to the Analytical Methods

We have highlighted that medium–low-power approaches (75x–145x) performed with metallurgical microscope, Dino-Lite microscope, or stereomicroscope are suitable for analyzing assemblages from high-energy contexts (e.g., water channels) (Bustos-Pérez & Ollé, 2024; Bustos-Pérez et al., 2019; Chambers, 2005; Hosfield & Chambers, 2005; Petraglia & Potts, 1994; Schick, 1986; Shackley, 1974, 1975), but are inadequate for low-entropy contexts. In such cases, high magnifications (≥200x) under reflected light microscope (digital or metallurgical) are essential to detect weakly developed post-depositional polishes and subtle edge modifications (Burroni et al., 2002; Donahue et al., 2019). SEM, despite high resolution, is less effective. This limitation is due not only to the grayscale nature of SEM images (see Fig. 1 in Chu et al., 2015) but also to the variability introduced by different coating materials, electron energies, and spot sizes, which can generate minor yet significant inconsistencies in image quality (Ollé et al., 2016; Vander Voort, 1986). Additionally, key microwear features such as micro-striations or micro-pitting are often difficult to discern with SEM, while they are readily visible under metallurgical microscopy (Borel et al., 2014; Ollé et al., 2016; Galland et al., 2025). Reflected light microscopy remains optimal for assessing surface reflectivity, polish coalescence, and other subtle attributes, enabling differentiation of post-depositional polish development stages (Supplementary Material 1, Fig. S6). Confocal microscopy effectively documents micro-topographical differences between severely altered artifacts and across preservation states (Galland et al., 2019, Fig. 5, p.50). Polish visibility improves with fine-texture mapping (Donahue et al., 2019, Fig. 4B, p.543), though grayscale rendering limits polish characterization compared to metallurgical microscopy.

## Conclusion

This review identifies and examines four key methodological challenges in using micro-alteration traces on flint artifacts to investigate archaeological site formation processes.

Our review of the post-depositional polish debate reveals persistent disagreement regarding their mechanical versus chemical origins, highlighting how limited mineralogical understanding has impeded the development of a typology based on their formative processes. While specific formation processes (e.g., wind abrasion and freeze-thaw cycles) have been seldomly identified, the underlying tribological mechanisms (mechanical, chemical, or combined) remain unclear. Mineralogical characterization using infrared spectroscopy to detect potential amorphous silica films would enable differentiation between mechanical, chemical, and combined formation processes. Only upon clarifying these aspects can a coherent typology of post-depositional polishes—based on their underlying processes—be established.

Classifying lithic assemblages by degree of surface and ridge alteration provides valuable insights. High-power analysis (≥200x) has been applied to assemblages belonging to “primary deposition” sites, whether assessing ridge rounding or micro-surface abrasion (Bradley et al., 1993; Vallin et al., 2001; Discamps et al., 2019; Donahue et al., 2019; Baillet et al., 2025; Bachellerie et al., submitted). Other researchers have applied medium-low magnifications (75x–145x) to “secondary deposition” assemblages (Shackley, 1974, 1975; Knutsson and Lindé, (Knutsson 1990); Hosfield & Chambers, 2005; Chambers, 2005; Bustos-Pérez et al., 2019). Current micro-surface roughness quantification methods have only been applied to small subsets of assemblages for methodological validation, without broader taphonomic applications. We recommend future studies implement systematic ascending ordinal classification of micro-alteration intensity (i.e., both micro-surfaces and ridges) to advance understanding of site formation processes.

For primary deposition sites—where post-depositional polishes may be subtle—microwear analyses addressing site formation processes require examination of thoroughly clean micro-surfaces. Although defining a “perfectly clean” surface remains subjective, our case study from Les Cottés demonstrates that initial assessments under high-magnification microscopy should be interpreted cautiously. Surfaces appearing to exhibit post-depositional polish after mild cleaning may retain extraneous residues, masking underlying features. We therefore recommend testing intensive cleaning protocols on unused waste debitage flakes and subsequently adjust the cleaning procedure of the whole sample accordingly, ensuring optimal surface preparation for taphonomic investigations.

Reproducing the precise conditions and effects of key post-depositional phenomena—particularly their characteristic microwear signatures—is crucial for developing robust methodological frameworks. Our review highlights that low-entropy contexts, especially important for behavioral archaeology, remain experimentally understudied. Among relevant processes, gentle cryoturbation (with centimeter-scale vertical displacements or more) and frictionless chemical alterations present persistent methodological challenges. We suggest targeted, controlled in vitro experiments to artificially accelerate and study these complex processes and their micro-alteration features.

Our review also shows that developing a typology of natural micro-polishes corresponding to specific post-depositional processes (e.g., aeolian abrasion, water run-off, and freeze-thaw cycles) through experimental reproduction has achieved partial success over the past fifty years (Stapert, 1976; Plisson & Mauger, 1988; Knutsson & Linde, 1990; Caspar et al., 2003; Asryan et al., 2017; Michel et al., 2019; Baillet et al., 2023, 2025; Galland et al., 2025). Crucially, all qualitative descriptors of these polishes—including coalescence, smoothness/roughness, reflectivity, directionality, micro-pits (diameter ≥1 µm), craters (diameter ≥10 µm), and micro-striations—must be documented at high magnifications (≥200x) using metallurgical microscopy. Furthermore, quantitative roughness parameters obtained through confocal microscopy are equally vital for defining and differentiating specific post-depositional polish types (Galland et al., 2019).

After addressing the challenges that require improvement, this article has also demonstrated that micro-traceology is already moving toward the new avenues outlined in this review. We believe that these methodological advancements, currently isolated and marginal, should be integrated as routine practices by most microwear analysts. Ultimately, this effort is about strengthening the initial stage of functional analysis in lithic assemblages, ensuring that the reflected light (metallurgical or digital) microscope—traditionally used for this purpose—can simultaneously be employed for both taphonomic examination (including surface alteration and ridge width) and functional study, without significantly extending the overall time required for analysis. Through these collective efforts, micro-traceology is poised to become an important analytical tool in investigations concerning the taphonomy of prehistoric sites.

## Supplementary Information

Below is the link to the electronic supplementary material.

Supplementary file1 (DOC 35221 KB)

## Data Availability

No datasets were generated or analysed during the current study.

## References

[CR1] Akoshima, K. (1987). Microflaking quantification. In G. Sieveking, & M. H. Newcomer (Eds.), *The human uses of flint and chert: Proceedings of the Fourth International Flint Symposium held at Brighton Polytechnic* (pp. 71–79). Cambridge University Press.

[CR2] Anderson-Gerfaud, P. (1981). *Contribution méthodologique à l'analyse des microtraces d'utilisation sur les outils préhistoriques*. PhD dissertation,. University of Bordeaux.

[CR3] Ascher, R. (1968). *Time’s arrow and the archaeology of a contemporary community* (pp. 47–79). Settlement Archaeology, Palo Alto, National Press Books.

[CR4] Asryan, L., Ollé, A., Moloney, N., King, T., & Murray, J. (2017). Chemical alteration of lithic artifacts: An experimental case study on the effect of guano on stone flakes and its contextualization in the archaeological assemblage of Azokh cave (Southern Caucasus). *Archaeometry,**59*(6), 981–999. 10.1111/arcm.12300

[CR5] Aubry, M. P., Dewolf, Y., & Muxart, T. (1975). Altération des silex de la craie : étude expérimentale : premières observations. *Comptes Rendus De L’académie des Sciences De Paris,**280*, 1509–1512.

[CR6] Baesemann, R. (1986). Natural alterations of stone artifact materials. In L. R. Owen & G. Unrath (Eds.), *Technical aspects of microwear studies on stone tools, Tübingen, February 1985* (pp. 97–102). Early Man News.

[CR7] Baillet, M., Chen, H., & Li, Y., (2023). Addressing the issue of “pitted” aspects of obsidian surface at Dadong site, China. A preliminary experimental approach. Poster presented at the UISPP XX° World Congress, Timişoara (Romania).

[CR8] Baillet, M., Vallin, L., Caspar, J. P., & Masson, B., (2025). The contribution of microwear analysis to a detailed reconstruction of the taphonomy of lithic assemblages in primary context : The example of Hermies le Champ Bruquette (Northern France). 10.2139/ssrn.5124331

[CR9] Bertran, P., Todisco, D., Bordes, J. G., Discamps, E., & Vallin, L. (2019). Perturbation assessment in archaeological sites as part of the taphonomic study: A review of methods used to document the impact of natural processes on site formation and archaeological interpretations. *Paleo,**30*(1), 52–75. 10.4000/paleo.4378

[CR10] Beyries, S., Delamare, F., & Quantin, J.-C. (1988). Tracéologie et rugosimétrie tridimensionnelle. In Beyries, S. (Dir.), Industries lithiques : tracéologie et technologie. In *Proceedings of the symposium, Centre de Recherches Archéologiques du CNRS, Valbonne 18–20 October 1986* (pp. 115–132). BAR International Series, 411 (2).

[CR11] Blomberg, A., Hogmark, S., & Lu, J. (1993). An electron microscopy study of worn ceramic surfaces. *Tribology International,**26*(6), 369–381. 10.1016/0301-679X(93)90075-C

[CR12] Borden, F. W. (1971). The use of surface erosion observations to determine chronological sequence in artifacts from a Mojave desert. *Archaeological Survey Association of Southern California, 7*. 10.2307/279602

[CR13] Bordes, F. (1950). Du poli particulier de certains silex taillés. *L’Anthropologie,**54*, 161–163.

[CR14] Borel, A., Ollé, A., Vergès, J. M., & Sala, R. (2014). Scanning electron and optical light microscopy: Two complementary approaches for the understanding and interpretation of usewear and residues on stone tools. *Journal of Archaeological Science,**48*, 46–59. 10.1016/j.jas.2013.06.031

[CR15] Borras, T.R., (1990). Chemical process of cleaning in microwear studies: Conditions and limits of attack. Application to archaeological sites. In Graslund, B., Knutsson, H., Knutsson, K., Taffinder, J. & Stina, E. (Eds.), *The interpretative possibilities of microwear studies* (pp. 179–184). Societas Archaeologica Upsaliensis, 14.

[CR16] Borrazzo, K. B. (2006). Tafonomía lítica en dunas: Una propuesta para el análisis de los artifactos líticos. *Intersecciones En Antropología, 7*, 247–261.

[CR17] Boule, M. (1905). *L’origine des Éolithes. L’anthropologie,**26*, 237–267.

[CR18] Bradley, R., & Clayton, C. (1987). The influence of flint microstructure on the formation of micro-wear polishes. In G. Sieveking & M. Newcomer (Eds.), *The human uses of flint and chert* (pp. 81–89). Cambridge University Press.

[CR19] Bradley, R., Chowne, P., Cleal, R. M. J., Healy, F., & Kinnes, I. (1993). Excavations on Redgate Hill, Hunstanton, Norfolk, and at Tattershall Thorpe, Lincolnshire. In D. Buckley (Ed.), *East Anglian Archaeology, Norfolk Museums Service* (p. 57). Heritage Trust of Lincolnshire.

[CR20] Burroni, D.-B., Donahue, R.-E., Pollard, A.-R., & Mussi, M. (2002). The surface alteration features of flint artifacts as a record of environmental processes. *Journal of Archaeological Science, 29*, 1277–1287. 10.1006/jasc.2001.0771

[CR21] Bustos-Pérez, G., & Ollé, A. (2024). The quantification of surface abrasion on flint stone tools. *Archaeometry, 66*(2), 247–265. 10.1111/arcm.12913

[CR22] Bustos-Pérez, G., Díaz, S., & Baena, J. (2019). An experimental approach to degrees of rounding among lithic artifacts. *Journal of Archaeological Method and Theory, 26*, 1243–1275. 10.1007/s10816-018-9409-8

[CR23] Caspar, J. P., Masson, B., & Vallin, L. (2003). Poli de bois ou poli de glace au Paléolithique inférieur et moyen ? Problèmes de convergence taphonomique et fonctionnelle. *Bulletin de la Société Préhistorique Française, 100*(3), 453–462. 10.3406/bspf.2003.12866

[CR24] Caspar, J. P., Masson, B., & Vallin, L. (2009). Taphonomie des ensembles lithiques du Paléolithique moyen en contexte loessique. *L’approche Expérimentale. Les Nouvelles De L’archéologie, 118*, 21–26. 10.4000/nda.882

[CR25] Caux, S., Galland, A., Queffelec, A., & Bordes, J. G. (2018). Aspects and characterization of chert alteration in an archaeological context: A qualitative to quantitative pilot study. *Journal of Archaeological Science: Reports, 20*, 210–219. 10.1016/j.jasrep.2018.04.027

[CR26] Chambers, J. (2005). Like a rolling stone? The identification of fluvial transport damage signatures on secondary context bifaces. *Lithics, 24*, 66–77.

[CR27] Christensen, M., Walter, P., & Menu, M. (1992). Usewear characterisation of prehistoric flints with IBA. *Nuclear Instruments and Methods in Physics Research, B64*, 488–493.

[CR28] Chu, W., & Hosfield, R. (2020). Lithic artifact assemblage transport and microwear modification in a fluvial setting: A radio frequency identification tag experiment. *Geoarchaeology,**35*, 591–608. 10.1002/gea.21788

[CR29] Chu, W., Thompson, C., & Hosfield, R. (2015). Micro-abrasion of flint artifacts by mobile sediments: A taphonomic approach. *Archaeological and Anthropological Sciences,**7*(1), 3–11. 10.1007/s12520-013-0157-0

[CR30] Clark, J. D., & Kleindienst, M. R. (1974). The Stone Age cultural sequence: Terminology, typology and raw material. In J. D. Clark (Ed.), *The Kalambo Falls prehistoric site. The Later Prehistoric Cultures* (pp. 71–106). Cambridge University Press.

[CR31] Claud, É. (2008). *Le statut fonctionnel des bifaces au Paléolithique moyen récent dans le Sud-Ouest de la France*. PhD dissertation, University of Bordeaux.

[CR32] Claud, E., & Bertran, P. (2010). Effet de la solifluxion sur les traces d’utilisation des outils lithiques : mise en place d’une expérimentation in vivo à Gavarnie (Hautes-Pyrénées, France). In M. P. Coumont, C. Thiébaut, & A. Averbouh (Eds.), *Sharing taphonomic approaches, workshop n° 16 - XVe International Congress UISPP, Lisboa, Septembre 2006* (pp. 31–42). Paleo, 3.

[CR33] Clemente-Conte, I. (1997). *Los instrumentos líticos de Túnel VII: una aproximación etnoarqueológica*. Treballs d’Etnoarqueologia, 2.

[CR34] Coffey, B. P. (1994). The chemical alteration of microwear polishes: An evaluation of the Plisson and Mauger findings through replicative experimentation. *Lithic Technology,**19*(2), 88–92. 10.1080/01977261.1994.11720914

[CR35] Copeland, L. (1989). Analysis of the paleolithic artifacts from the sounding of A. Garrard at C-Spring, 1985 season. In L. Copeland & F. Hours (Eds.), *The hammer on the rock: Studies in the early Palaeolithic of Azraq, Jordan (Part 2)* (pp. 329–390). British Archaeological Reports International, 540.

[CR36] Davis, E. L. (1967). Man and water at Pleistocene lake Mohave. *American Antiquity, 32*(3), 345–353.

[CR37] Deprez, M., Kock, T., Schutter, G., & Gnubble, V. (2020). A review on freeze-thaw action and weathering of rocks. *Earth-Science Reviews,**203*, 1–20. 10.1016/j.earscirev.2020.103143

[CR38] Discamps, E., Bachellerie, F., Baillet, M., & Sitzia, L. (2019). The use of spatial taphonomy for interpreting Pleistocene palimpsests: An interdisciplinary approach to the Châtelperronian and carnivore occupations at Cassenade (Dordogne, France). *PaleoAnthropology*, 362–388. 10.4207/PA.2019.ART136

[CR39] Donahue, R. E., & Burroni, D. B. (2004). Lithic microwear analysis and the formation of archaeological assemblages. In E. A. Walker, F. Wenban-Smith, & F. Healy (Eds.), *Lithics in action: Papers for the conference lithic studies in the year 2000* (pp. 140–148). Oxbow.

[CR40] Donahue, R. E., Fischer, A., Burroni, D. B., Malm, T., & Johansen, M. (2019). Microwear analysis aiding excavation prioritization at the submerged Mesolithic settlement of Orehoved, Denmark. *Journal of Archaeological Science: Reports,**23*, 540–548. 10.1016/j.jasrep.2018.11.009

[CR41] Engel, C. G., & Sharp, R. P. (1958). Chemical data on desert varnish. *Bulletin of the Geological Society of America,**69*, 487–518. 10.1130/0016-7606(1958)69[487:CDODV]2.0.CO

[CR42] Eren, M. I., Boehm, A. R., Morgan, B. M., Anderson, R., & Andrews, B. (2011). Flaked stone taphonomy: A controlled experimental study of the effects of sediment consolidation on flake edge morphology. *Journal of Taphonomy,**9*(3), 201–217.

[CR43] Evans, A.-A., & Donahue, R.-E. (2005). The elemental chemistry of lithic microwear: An experiment. *Journal of Archaeological Science,**32*, 1733–1740. 10.1016/j.jas.2005.06.010

[CR44] Fiers, G., Halbrucker, E., Kock, T. D., Laforce, B., Vandendriessche, H., Messiaen, L., Vincze, L., Crombé, P., & Cnudde, V. (2018). Preliminary characterization of flint raw material used on prehistoric sites in NW Belgium. *Geoarcheology,**34*, 400–412. 10.1002/gea.21719

[CR45] Galland, A., Clemente-Conte, I., Boisserie, J. R., & Delagnes, A. (2025). *How Depositional Environments Impact the Microwear Preservation of Quartz Artifacts: Insights from the Oldowan of the Shungura Formation (Ethiopia).*10.31233/osf.io/9k8p3

[CR46] Galland, A., Queffelec, A., Caux, S., & Bordes, J. G. (2019). Quantifying lithic surface alterations using confocal microscopy and its relevance for exploring the Châtelperronian at La Roche-à-Pierrot (Saint-Césaire, France). *Journal of Archaeological Science,**104*, 45–55. 10.1016/j.jas.2019.01.009

[CR47] Gauthier, G., & Burke, A. L. (2011). The effects of surface weathering on the geochemical analysis of archaeological lithic samples using non-destructive polarized energy dispersive XRF. *Geoarchaeology,**26*(2), 269–291.

[CR48] Glauberman, P. J., & Thorson, R. M. (2012). Flint patina as an aspect of “flaked stone taphonomy”: A case study from the loess terrain of the Netherlands and Belgium. *Journal of Taphonomy,**10*(1), 21–43.

[CR49] Goffer, Z. (1980). *Archaeological chemistry*. Wiley-Interscience. 10.1002/0471915254

[CR50] Goren-Inbar, N., Belitzky, S., Goren, Y., Rabinovich, R., & Saragusti, I. (1992). Gesher Benot Ya’aqov-the “Bar”: An Acheulian assemblage. *Geoarchaeology,**7*, 27–40. 10.1002/gea.3340070103

[CR51] Harding, P., Gibbard, P. L., Lewin, J., Macklin, M. G., & Moss, E. H. (1987). The transport and abrasion of flint hand axes in a gravel bed river. In G. Sieveking & M. Newcomer (Eds.), *The human uses of flint and chert: Papers from the Fourth International Flint Symposium, Proceedings of the fourth international flint symposium held at Brighton Polytechnic* (pp. 115–126). Cambridge University Press.

[CR52] Herdianita, N. R., Browne, P. R. L., Rodgers, K. A., & Campbell, K. A. (2000). Mineralogical and textural changes accompanying ageing of silica sinter. *Mineralium Deposita,**35*, 48–62. 10.1007/s001260050005

[CR53] Hiscock, P. (1985). The need for a taphonomic perspective in stone artifact analysis. *Queensland Archaeological Research*, *2*, 82–95. 10.25120/qar.2.1985.197

[CR54] Honea, K. (1964). The patination of stone artifacts. *Plains Anthropologist,**9*, 14–17. 10.1080/2052546.1964.11908372

[CR55] Hooke, R. L., Yang, H. Y., & Weiblen, P. W. (1969). Desert varnish: An electron probe study. *Journal of Geology,**77*, 275–288. 10.1086/627435

[CR56] Hosfield, R. T., & Chambers, J. C. (2005). River gravels and flakes: New experiments in site formation, stone tool transportation and transformation. In M. Fansa (Ed.), *Experimentelle Archäologie in Europa, Bilanz 2004* (pp. 57–74). Isensee Verlag.

[CR57] Howard, C. D. (1999). Amorphous silica, soil solutions, and archeological flint gloss. *North American Archaeologist,**20*(209), 215. 10.2190/68LF-CM3F-GGHR-L18P

[CR58] Howard, C. D. (2002). The gloss patination of flint artifacts. *Plains Anthropologist,**47*(182), 283–288. 10.1080/2052546.2002.11932098

[CR59] Hue, E. (1929). Recherches sur la Patine des Silex. *Bulletin de la Société Préhistorique Française,**26*, 461–468.

[CR60] Ibañez, J. J., & Mazzucco, N. (2021). Quantitative use-wear analysis of stone tools: Measuring how the intensity of use affects the identification of the worked material. *PLoS One,**16*(9), Article e0257266. 10.1371/journal.pone.0257266 34543297 10.1371/journal.pone.0257266 PMC8452086

[CR61] Kaminska, J., Mycielska-Dowgiallo, E., & Szymczar, K. (1993). Post-depositional changes on surfaces of flint artifacts as observed under a scanning electron microscope. In P. Anderson, S. Beyries, M. Otte, & H. Plisson (Eds.), *Traces et fonctions: les gestes retrouvés. Vol. 2. Actes du Colloque international de Liège, 8–9–10 décembre 1990* (pp. 467–476). E.R.A.U.L., 50.

[CR62] Keeley, L. -H. (1980). *Experimental determination of stone tool uses*. The University of Chicago Press.

[CR63] Knutsson, K. (1988). Chemical etching of wear features on experimental quartz tools. In S. L. Olsen (Ed.), *Scanning electron microscopy in archaeology* (pp. 117–153). British Archaeological Report International Series, 452.

[CR64] Knutsson, K., & Linde, K. (1990). Post-depositional alterations of wear marks on quartz tools. Preliminary observations of an experiment with Aeolian abrasion. In M. R. Séronie-Vivien & M. Lenoir (Eds.), *Le silex de sa genèse à l'outil, Ve International symposium on flint* (pp. 607–618). Cahiers du Quaternaire, 17.

[CR65] Lenoble, A. (2005). Ruissellement et formation des sites préhistoriques: référentiel actualiste et exemples d’application au fossile. In *British archaeological report international series* (p. 1363).

[CR66] Lenoble, A., Bertran, P., & Lacrampe, F. (2008). Solifluction-induced modifications of archaeological levels: Simulation based on experimental data from a modern periglacial slope and application to French Palaeolithic sites. *Journal of Archaeological Science,**35*, 99–110. 10.1016/j.jas.2007.02.011

[CR67] Levi-Sala, I. (1986a). Use wear and post-depositional surface modification: A word of caution. *Journal of Archaeological Science,**13*, 229–244. 10.1016/0305-4403(86)90061-0

[CR68] Levi-Sala, I. (1986b). Experimental replication of post-depositional surface modification on chert. In L. Owen & G. Unrath (Eds.), *Technical aspects of microwear studies on stone tools* (pp. 103–109). Early Man News.

[CR69] Levi-Sala, I. (1988). Processes of polish formation on flint tool surfaces. Industries lithiques: Tracéologie et technologie. *British Archaeological Reports International Series,**411*, 83–97.

[CR70] Lhomme, V., Nicoud, E., Chaussé, C., & Coudenneau, A. (2010). Estimation du degré de cohérence d’un ensemble archéologique du Paléolithique moyen récent en contexte fluviatile. L’exemple du niveau D2 du site du Fond-des-Blanchards à Gron (Yonne, France). In M. P. Coumont, C. Thiébaut, & A. Averbouh (Eds.), *Sharing taphonomic approaches, workshop n° 16 - XVe International Congress UISPP, Lisboa, Septembre 2006* (pp. 53–63). Paleo, 3.

[CR71] Macdonald, D. A., & Evans, A. A. (2014). Evaluating surface cleaning techniques of stone tools using laser scanning confocal microscopy. *Microscopy Today,**22*(3), 22–27. 10.1017/S1551929514000364

[CR72] Mansur, M. E. (1982). Microwear analysis of natural and use striations : New clues to the mechanisms of striation formation. *Studia Praehistorica Belgica,**2*, 213–233.

[CR73] Mansur, M. E. (1986). Microscopie du matériel lithique préhistorique : traces d'utilisation, altérations naturelles, accidentelles et technologiques. In *Exemples de Patagonie. CNRS Editions*. Cahiers du Quaternaire, 9.

[CR74] Marreiros, J. M., Gibaja Bao, J. F., & Bicho, N. F. (2015). Use-wear and residue analysis in archaeology. In C. E. Orser & M. B. Schiffer (Eds.), *Manuals in archaeological method, theory and technique*.

[CR75] Masson, A. (1981). Altérations des silex préhistoriques : dissolution, néogenèse siliceuse, implications sédimentologiques. *Comptes Rendus De L’académie des Sciences, Série II,**292*, 1533–1537. 10.4000/quaternaire.6658

[CR76] Masson, A., Coqueugniot, E., & Roy, S. (1981). Silice et traces d’usage: Le lustré des faucilles. *Publications Du Musée des Confluences,**19*, 43–52.

[CR77] Mazzucco, N., Trenti, F., Clemente Conte, I., & Gibaja Bao, J. F., (2013). Chert taphonomical alterations: Preliminary experiments. In A. Palomo Pérez, R. Piqué i Huerta, X. & Terradas Batlle (Eds.), Experimentación en arqueología estudio y difusión del pasado. *International Congress of Experimental Archaeology, Banyoles, Serie Monografica del Museo de Arqueología de Cataluña**2*(25), 269–277.

[CR78] McPherron, S. J. P., Braun, D. R., Dogandžić, T., Archer, W., Desta, D., & Lin, S. C. (2014). An experimental assessment of the influences on edge damage to lithic artifacts: A consideration of edge angle, substrate grain size, raw material properties, and exposed face. *Journal of Archaeological Science,**49*, 70–82. 10.1016/j.jas.2014.04.003

[CR79] Meeks, N. D., Sieveking, G., Tite, M. S., & Cook, J. (1982). Gloss and use-wear traces on flint sickles and similar phenomena. *Journal of Archaeological Science,**9*, 317–340. 10.1016/0305-4403(82)90038-3

[CR80] Meillet, A. (1866). Recherches chimiques sur la patine des silex taillés. *Moniteur De L’archéologue,**2*(1), 247–254.

[CR81] Michel, M., Cnuts, D., & Rots, V. (2019). Freezing in-sight: The effect of frost cycles on use-wear and residues on flint tools. *Archaeological and Anthropological Sciences,**11*, 5423–5443. 10.1007/s12520-019-00881-w

[CR82] Moncel, M. H., Lemorini, C., Eramo, G., Fioretti, G., Daujeard, C., Curci, A., Berto, C., Hardy, B., Pineda, A., Rineau, V., Carpentieri, M., Sala, B., Arzarello, M., Mecozzi, B., Iannucci, A., Sardella, R., & Piperno, M. (2023). A taphonomic and spatial distribution study of the new levels of the middle Pleistocene site of Notarchirico (670–695 ka, Venosa, Basilicata, Italy). *Archaeological and Anthropological Sciences,**15*(106), 2–38. 10.1007/s12520-023-01809-1

[CR83] Morey, G. W., Fournier, R. D., & Rowe, J. J. (1964). The solubility of amorphous silica at 25°C. *Journal of Geophysical Research,**69*, 1995–2002. 10.1029/JZ069i010p01995

[CR84] Moses, C., Robinson, D., & Barlow, J. (2014). Methods for measuring rock surface weathering and erosion: A critical review. *Earth-Science Reviews,**135*, 141–161. 10.1016/j.earscirev.2014.04.006

[CR85] Moss, E. -H. (1983). *The functional analysis of flint implements. Pincevent and Pont-d'Ambon, two case studies from the french final Paleolithic* (Vol. 177). BAR International Series.

[CR86] Ollé, A., Pedergnana, A., Fernandez-Marchena, J. L., Martin, S., Borel, A., & Aranda, V. (2016). Microwear features on vein quartz, rock crystal and quartzite: A study combining optical light and scanning electron microscopy. *Quaternary International,**424*, 154–170. 10.1016/j.quaint.2016.02.005

[CR87] Pawlikowski, M., & Wasilewski, M. (2002). Mineralogical investigation of desert patina on flint artifacts: A case study. *Mediterranean Archaeology and Archaeometry,**2*(2), 23–34.

[CR88] Pedergnana, A., Calandra, I., Bob, K., Gneisinger, W., Paixao, E., Schunk, L., Hildebrandt, A., & Marreiros, J. (2020). Evaluating the microscopic effect of brushing stone tools as a cleaning procedure. *Quaternary International, 569-570*, 263–276.

[CR89] Pei, W. C. (1936). Le rôle des phénomènes naturels ans l’éclatement et le façonnement des roches dures utilisées par l’homme préhistorique. *Revue De Géographie Physique Et De Géologie Dynamique,**9*(4), 1–78.

[CR90] Perry, R. S., & Adams, J. B. (1978). Desert varnish: Evidence for cyclic deposition of manganese. *Nature,**276*, 489–491. 10.1038/276489a0

[CR91] Petraglia, M. D., & Potts, R. (1994). Water flow and the formation of Early Pleistocene artifact sites in Olduvai Gorge, Tanzania. *Journal of Anthropological Archaeology,**13*, 228–254. 10.1006/jaar.1994.1014

[CR92] Phillips, N., Pargeter, J., Low, M., & Mackay, A. (2019). Open-air preservation of miniaturised lithics: Experimental research in the Cederberg Mountains, southern Africa. *Archaeological and Anthropological Sciences,**11*, 5851–5877. 10.1007/s12520-018-0617-7

[CR93] Plisson, H. (1985). *Étude fonctionnelle d'outillages lithiques préhistoriques par l'analyse des micro-usures : recherche méthodologique et archéologique*. PhD dissertation, University of Paris I.

[CR94] Plisson, H., & Mauger, M. (1988). Chemical and mechanical alteration of microwear polishes: An experimental approach. *Helinium,**28*(1), 3–16.

[CR95] Potter, R. M., & Rossman, G. R. (1977). Desert varnish: The importance of clay minerals. *Science,**196*, 1446–1449. 10.1126/science.196.4297.1446 17776923 10.1126/science.196.4297.1446

[CR96] Prezzi, M., Monteiro, P. J. M., & Sposito, G. (1997). The alkali-silica reaction, part I: Use of the double-layer theory to explain the behavior of reaction-product gels. *ACI Materials Journal,**94*, 10–17.

[CR97] Rose, J. I., Hilbert, Y. H., Usyk, V. I., Bebber, M. R., Beshkani, A., Buchanan, B., Cascalheira, J., Chlachula, D., Dellmour, R., Eren, M. I., Garba, R., Hallinan, E., Li, L., Walker, R. S., & Marks, A. E. (2025). Mapping lateral stratigraphy at Palaeolithic surface sites: A case study from Dhofar. *Oman. Journal of Archaeological Science.,**173*, Article 106117. 10.1016/j.jas.2024.106117

[CR98] Rots, V. (2010). *Prehension and hafting traces on flint tools : A methodology*. University Press. 10.2307/j.ctt9qf05s

[CR99] Rottländer, R. (1975). The formation of patina on flint. *Archaeometry,**17*(1), 106–110. 10.1111/j.1475-4754.1975.tb00120.x

[CR100] Santonja, M., & Pérez-González, A. (2001). Lithic artifacts from the lower levels of Ambrona (Spain) taphonomic features. In G. Cavaretta, P. Gioia, M. Mussi, & M. R. Palomo (Eds.), *The world of elephants, international congress, Rome* (pp. 592–596). Consiglio Nazionale delle Ricerche.

[CR101] Schick, K. D. (1986). Stone age sites in the making: Experiments in the formation and transformation of archaeological occurrences. *British Archaeological Report International Series, 319*.

[CR102] Schiffer, M. B. (1987). *Formation processes of the archaeological record*. University of New Mexico Press.

[CR103] Schmidt, P., Rodriguez, A., Yanamandra, K., Behera, R. K., & Iovita, R. (2020). The mineralogy and structure of use-wear polish on chert. *Scientific Reports, 10*, 21512. 10.1038/s41598-020-78490-0 33299032 10.1038/s41598-020-78490-0 PMC7725782

[CR104] Schoville, B. J. (2010). Frequency and distribution of edge damage on Middle Stone Age lithic points, Pinnacle Point 13B, South Africa. *Journal of Human Evolution,**59*, 378–391. 10.1016/j.jhevol.2010.07.015 20934092 10.1016/j.jhevol.2010.07.015

[CR105] Sellier, D., & Stephant, N. (2017). Submicrometric metrology applied to SEM characterization of erosion processes on quartz and quartzites. *Géomorphologie : Relief, Processus, Environnement,**23*(1), 55–81. 10.4000/geomorphologie.11660

[CR106] Semenov, S. -A. (1964). *Prehistoric technology: An experimental study of the oldest tools and artifacts from traces of manufacture and wear*. Cory, Adams et Mackay.

[CR107] Shackley, M. S. (1974). Stream abrasion of flint implements. *Nature,**248*(5448), 501–502. 10.1038/248501a0

[CR108] Shackley, M. S. (1975). *A study of the Mousterian of Acheulian tradition industries of Southern Britain*. Unpublished Thesis dissertation, University of Southampton.

[CR109] Shea, J. J. (1999). Artifact abrasion, fluvial processes, and “living floors” from the Early Paleolithic site of Ubeidiya (Jordan Valley, Israel). *Geoarchaeology,**14*(2), 191–207. 10.1002/(SICI)1520-6548(199902)14:2<191::AID-GEA4>3.0.CO;2-K

[CR110] Shea, J., & Klenck, J. D. (1993). An experimental investigation of the effects of trampling on the results of lithic microwear analysis. *Journal of Archaeological Science,**20*, 175–194. 10.1006/jasc.1993.1013

[CR111] Shepherd, W. (1972). *Flint: Its origin, properties and uses*. Faber, Transatlantic Arts.

[CR112] Sieveking, G., & Clayton, C. J. (1986). Frost shatter and the structure of frozen flint. In G. Sieveking & M. B. Hart (Eds.), *The scientific study of flint, proceedings of the fourth international flint symposium held at Brighton Polytechnic, 10-15 April 1983* (pp. 283–290). Cambridge University Press.

[CR113] Singer, R., Wymer, J. J., Gladfelter, B. G., & Wolff, R. G. (1973). Excavation of the Clactonian industry at the golf course, Clacton-on-Sea, Essex. *Proceedings of the Prehistoric Society,**39*, 6–74. 10.1017/S0079497X00011610

[CR114] Stapert, D. (1976). Some natural surface modification on flint in the Netherlands. *Palaeohistoria,**18*, 8–14.

[CR115] Stein, J. K. (1987). Deposits for archaeologists. *Advances in Archaeological Method and Theory,**11*, 337–395.

[CR116] Stepanchuk, V., & Naumenko, O. (2024). Investigating the post-discard alteration of flint artifacts at Medzhibozh 1 Lower Palaeolithic Site, Ukraine. *Journal of Archaeological Science: Reports, 53*, 104374. 10.1016/j.jasrep.2024.104374

[CR117] Suratwala, T., Steele, W., Wong, L., Feit, M. D., Miller, P. E., Dylla-Spears, R., Shen, N., & Desjardin, R. (2015). Chemistry and formation of the Beilby layer during polishing of fused silica glass. *Journal of the American Ceramic Society,**98*(8), 2395–2402. 10.1111/jace.13659

[CR118] Taylor, A., Bernard-Guelle, S., Rué, M., Fernandes, P., & Chesnaux, L. (2021). Le site moustérien récent du fossé du Bois à Appoigny (Hameau des Bries, Vallée de l’Yonne): Approches taphonomique, pétrographique et techno-économique de l’industrie lithique. *Revue Archéologique de l’Est,**70*, 5–57.

[CR119] Texier, J. P., Bertran, P., Coutard, J. P., Francou, B., Gabert, P., Guadelli, J. L., Ozouf, J. C., Plisson, H., Raynal, J. P., & Vivent, D. (1998). TRANSIT, an experimental archaeological program in periglacial environment: Problem, methodology, first results. *Geoarchaeology,**13*, 433–473. 10.1002/(SICI)1520-6548(199806)13:5<433::AID-GEA1>3.0.CO;2-1

[CR120] Thiry, M., Fernandes, P., Milnes, A., & Raynal, J. P. (2014). Driving forces for the weathering and alteration of silica in the regolith: Implications for studies of prehistoric flint tools. *Earth-Science Reviews,**136*, 141–154. 10.1016/j.earscirev.2014.05.008

[CR121] Trauth, N., Vilas-Boas, G., Thiry, M., Badaut, D., & Eberhart, J. P. (1978). Silex et chailles du bassin de Paris: Modifications minéralogiques lors de leurs altérations. *Science Géological Bulletin,**31*(4), 173–183.

[CR122] Tringham, R., Cooper, G., Odell, G., Voytek, B., & Whitman, A. (1974). Experimentation in the formation of edge damage: A new approach to lithic analysis. *Journal of Field Archaeology, 1*(1/2), 171–196.

[CR123] Ugalde, P. C., Santoro, C. M., Gayo, E. M., Latorre, C., Maldonado, S., Pol-Holz, R. D., & Jackson, D. (2015). How do surficial lithic assemblages weather in arid environments? A case study from the Atacama Desert, Northern Chile. *Geoarchaeology,**30*, 352–368. 10.1002/gea.21512

[CR124] Vallin, L., Caspar, J. P., Guillemet, G., Masson, B., & Ozouf, J. C. (2013). Altérations des artifacts préhistoriques en silex par les processus périglaciaires: Présentation des expériences conduites au Centre de Géomorphologie du CNRS de Caen. *Quaternaire,**24*(3), 259–266. 10.4000/quaternaire.6658

[CR125] Vallin, L., Masson, B., & Caspar, J. P. (2001). Taphonomy at Hermies, France: A Mousterian knapping site in a loessic context. *Journal of Field Archaeology,**28*, 419–436. 10.1179/jfa.2001.28.3-4.419

[CR126] Vander Voort, G. F. (1986). The SEM as a metallographic tool. In G. F. Vander Voort (Ed.), *Applied metallography* (pp. 139–170). Springer. 10.1007/978-1-4684-9084-8_10

[CR127] Vaughan, P. (1981). Lithic microwear experimentation and the functional analysis of a Lower Magdalenian stone tool assemblage. PhD dissertation, University of Pennsylvania.

[CR128] Venditti, F., Tirillo, J., & Garcea, E. A. A. (2016). Identification and evaluation of post-depositional mechanical traces on quartz assemblages: An experimental investigation. *Quaternary International,**424*, 143–153. 10.1016/j.quaint.2015.07.046

[CR129] Vignard, E., & Vacher, G. (1964). Altération des silex paléolithiques de Nemours sous l’influence des climats qui se sont succédés du Périgordien Gravettien au Tardenoisien locaux. *Bulletin De La Société Préhistorique Française,**61*(1), 45–55.

[CR130] Villa, P., & Soressi, M. A. (2000). Stone tools in carnivore sites: The case of Bois Roche. *Journal of Anthropological Research,**56*, 187–215.

[CR131] Wang, Y., & Zhang, H. (2023). The synergic impacts of salt mixture and frost damage on rock decay: Implications for the deterioration of rock-hewn heritages. *Heritage Science,**11*(209), 1–16. 10.1186/s40494-023-01054-8

[CR132] Warren, S. H. (1905). On the origin of "eolithic" flints by natural causes, especially by the foundering of drift. *The Journal of the Anthropological Institute of Great Britain and Ireland*, *35*, 337–364. 10.2307/2843073

[CR133] Werner, J. J. (2018). An experimental investigation of the effects of post-depositional damage on current quantitative use-wear methods. *Journal of Archaeological Science: Reports,**17*, 597–604. 10.1016/j.jasrep.2017.12.008

[CR134] White, K., Bryant, R., & Drake, N. (1998). Techniques for measuring rock weathering: Application to a dated fan segment sequence in Southern Tunisia. *Earth Surface Processes and Landforms*, *23*, 1031–1043. 10.1002/(SICI)1096-9837(1998110)23:11<1031::AIDESP919>3.0.CO;2

[CR135] Yamada, S., & Sawada, A. (1993). The method of description for polished surfaces. In P. Anderson, S. Beyries, M. Otte, H. Plisson, (Dir.), *Traces et fonction : les gestes retrouvés, International symposium in Liege, 8-10 Décembre 1990* (pp. 447-457). ERAUL, 50 (2).

