# Supplementary material for: Let the Stones Shine: Assessing the Potential of Microwear Analysis on Flint Artifacts to Refine the Post-depositional History of Paleolithic Sites
Source: J Paleolit Archaeol. 2025 Aug 26;8(1):26. doi: 10.1007/s41982-025-00225-2 (PMC12378480; doi:10.1007/s41982-025-00225-2)
Supplement: Supplementary file 1 — Supplementary file1 (DOC 35221 KB) [file 41982_2025_225_MOESM1_ESM.doc]

**Supplementary material 1**


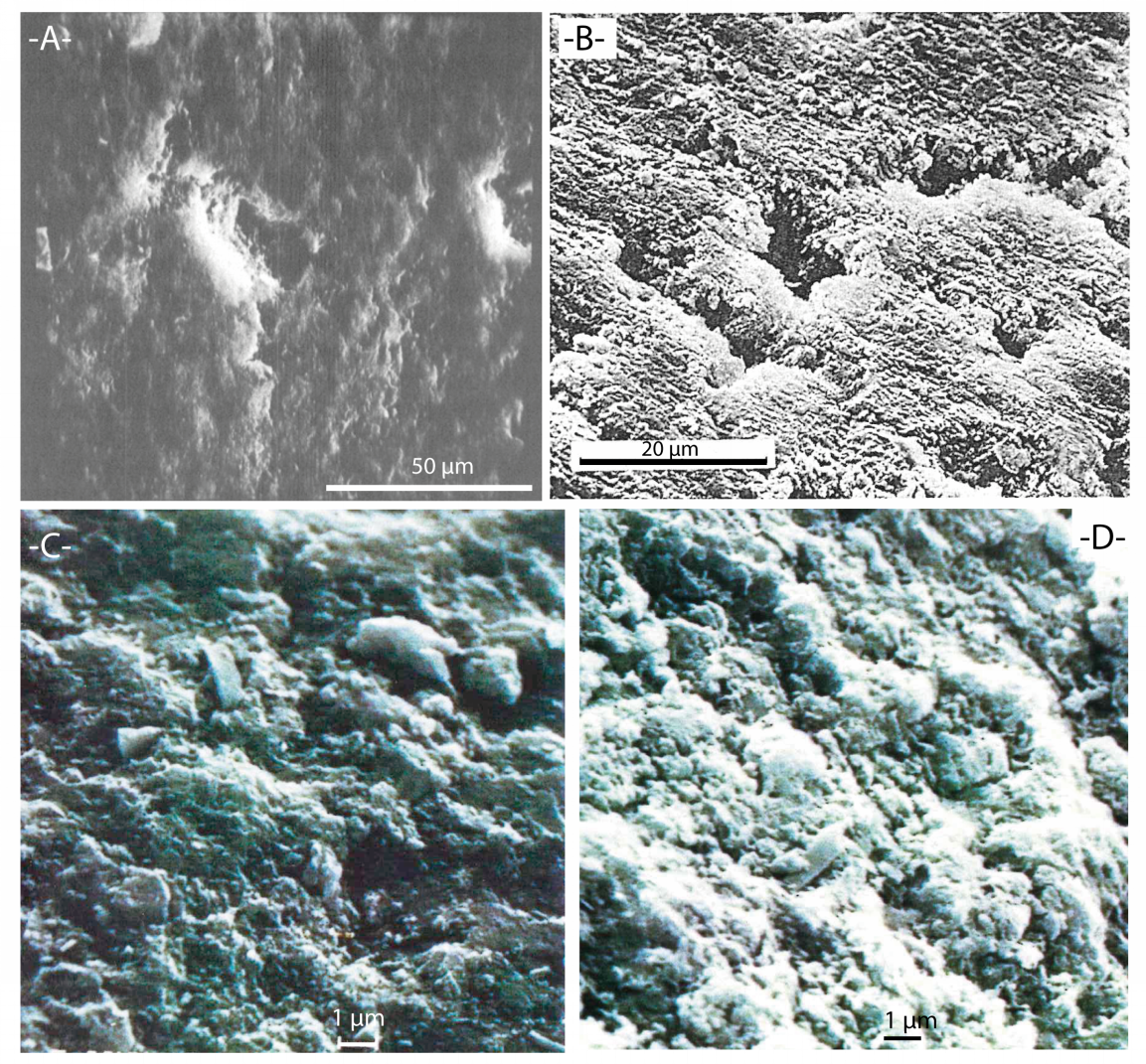


Fig. S1. Chemically altered experimental flint pieces, captured at very high magnifications under SEM, as published by different authors. -A-: plan-view of a chemically treated (undetermined conditions) micro-surface, showing «glossy patination and the start of the etched holes», each hole measuring about 10 µm (after Rottländer, 1975, plate 3, p.110, magn. 1040x) (N.B.: undetermined cleaning procedure after experiment). -B-: plan-view of the interior part of an intentionally cross-sectioned experimental flint after 15 freeze-thaw cycles, interpreted by the author as the «etching» of the interstitial chalcedony (highly soluble silica form) (Sieveking and Clayton, 1986, fig.32.2-F-, p. 286, magn. unknown) (N.B.: undetermined cleaning procedure after experiment). -C-: plan-view of an untreated surface of a freshly knapped chert (Tertiary lacustrine chert) (after Plisson, 1985, planche 9, p. 129, magn. 5000x). -D-: same piece (other area) after 285 hours of immersion in calcium hydroxide, interpreted by the author as showing no dissolution nor redeposition of silica, no etched hole and no white patina (after Plisson, 1985, planche 9, p. 129, magn. 5000x) (N.B.: cleaning procedure after experiment involved soaking few minutes in hot soapy water and acetone).


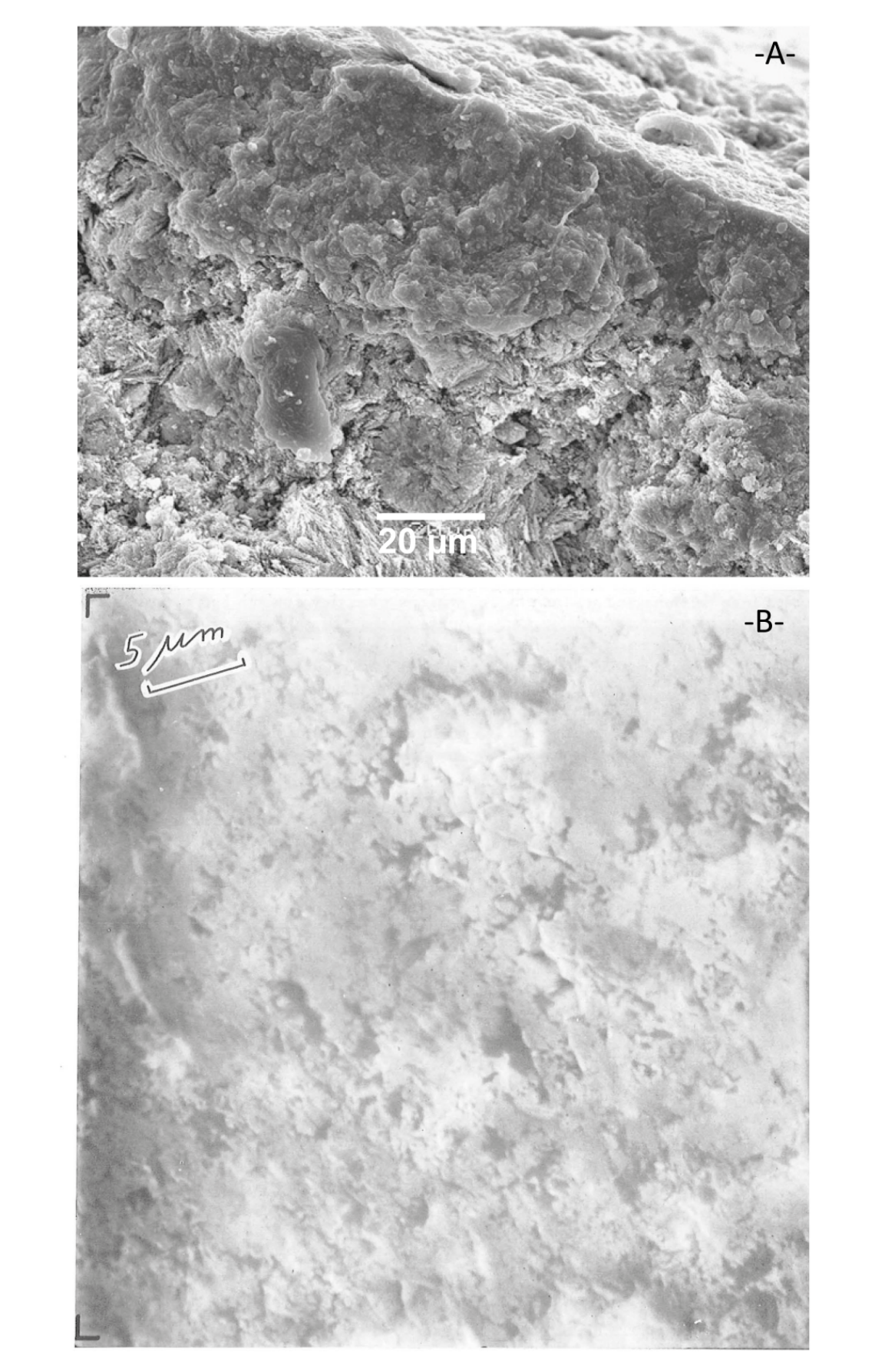


Fig. S2. Alterations on archaeological flint pieces, captured at very high magnifications under SEM, as published by different authors. -A-: intentionally cross-sectioned paleolithic artifact showing the inner part: above, on the edge of the fracture, the authors reported a thin layer «tightly cemented by secondary silica without any granulation», interpreted as the result of chemical dissolution and re-precipitation of silica. At the bottom of the image, they reported «the primary flint matrix in which the lengthening textures of chalcedony remain» (Thiry et al., 2014, fig.19, p.151, magn. 1000x) (N.B.: undetermined cleaning procedure). -B-: plan-view of a geological sample (collected in secondary position from sand dunes) micro-surface, showing a mirror polish, interpreted by the author as a combination between aeolian abrasion and chemical process (Stapert, 1976, fig.9, p. 19., magn. 3000x). (N.B.: undetermined cleaning procedure)


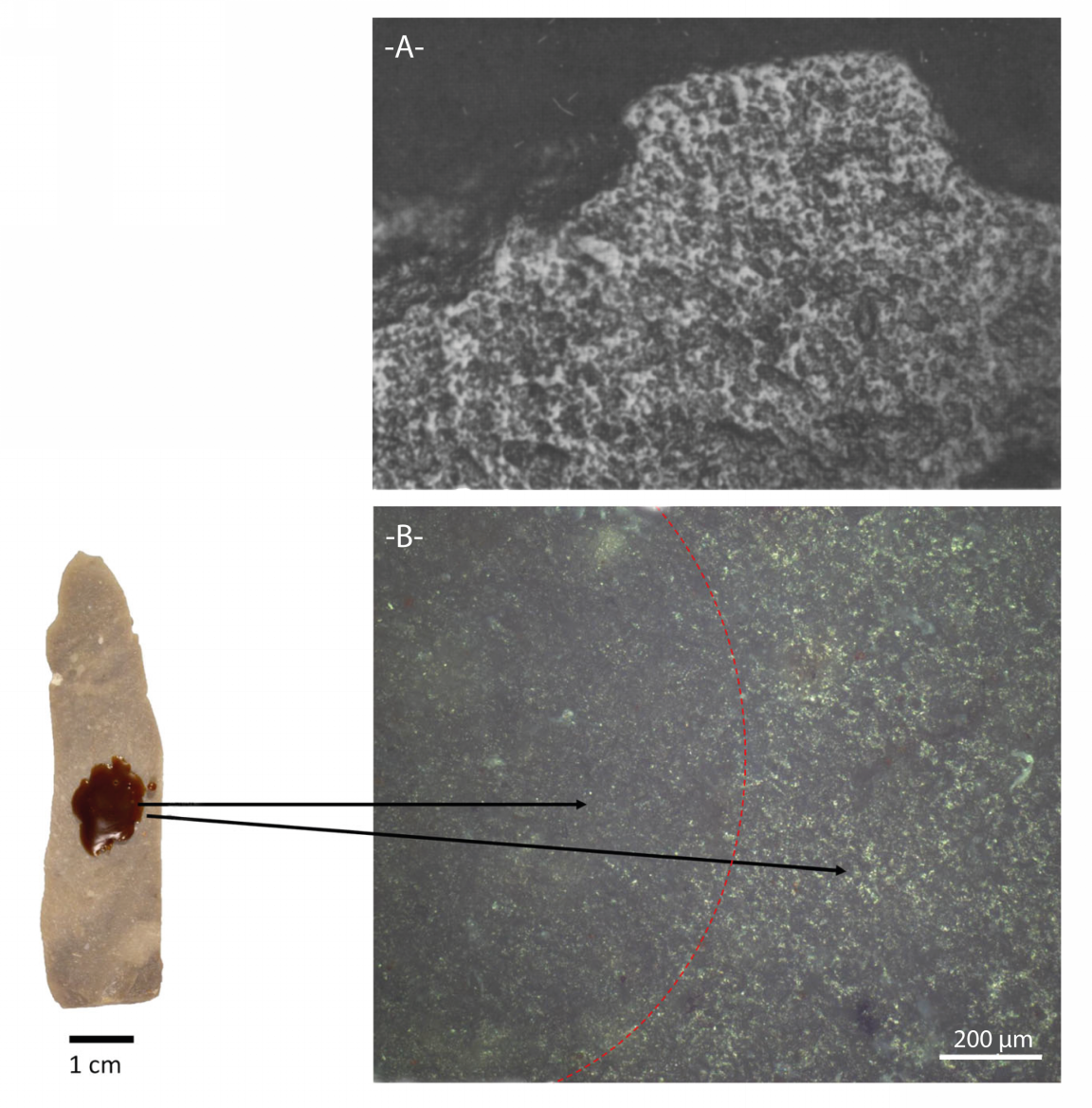


Fig. S3. Mechanically altered experimental flint pieces, captured at high magnifications under reflected light microscopes, as published by different authors. -A-: plan-view of the micro-surface after tumbling with wet gravelly sand for 50 hours, interpreted by the author as a polish of soil abrasion (Levi-Sala, 1986a, fig.3-e-, p. 236, magn. 200x). (N.B.: cleaning procedure involved chemical treatment and ultrasonic bath). -B-: plan-view of the micro-surface after freezing-thawing experience in loess for about 50 cycles, interpreted by the authors as a polish of soil abrasion (right part of the micrograph), while the glue prevented the surface from any polish (left part of the micrograph) (Michel et al., 2019, fig.25, magn. 200x). (N.B.: cleaning procedure involved chemical treatment and ultrasonic bath).


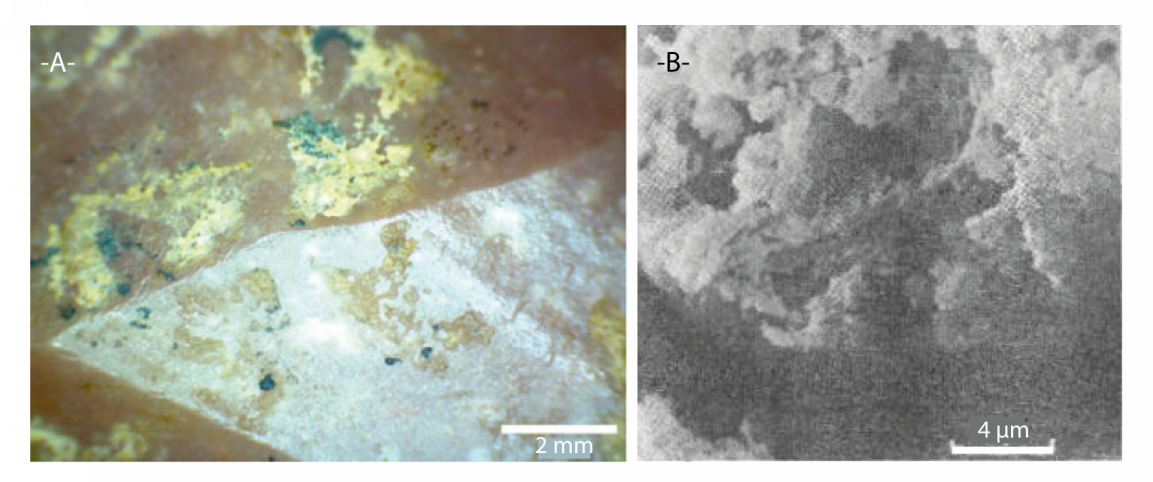


Fig.S4. Post-depositional dirty layer on archaeological flint artifacts, as published by different authors. -A-: macroscopic crust on paleolithic artifact surface from Azokh cave, before cleaning (Asryan et al., 2017, fig.6, p. 992, magn. 30x). -B-: microscopic layer on paleolithic artifact surface from Baume-Vallée site, interpreted by the author as clay cladding (Masson, 1981, fig. 1, n°4, p. 1535, magn. 5000x). (N.B.: undetermined cleaning procedure).


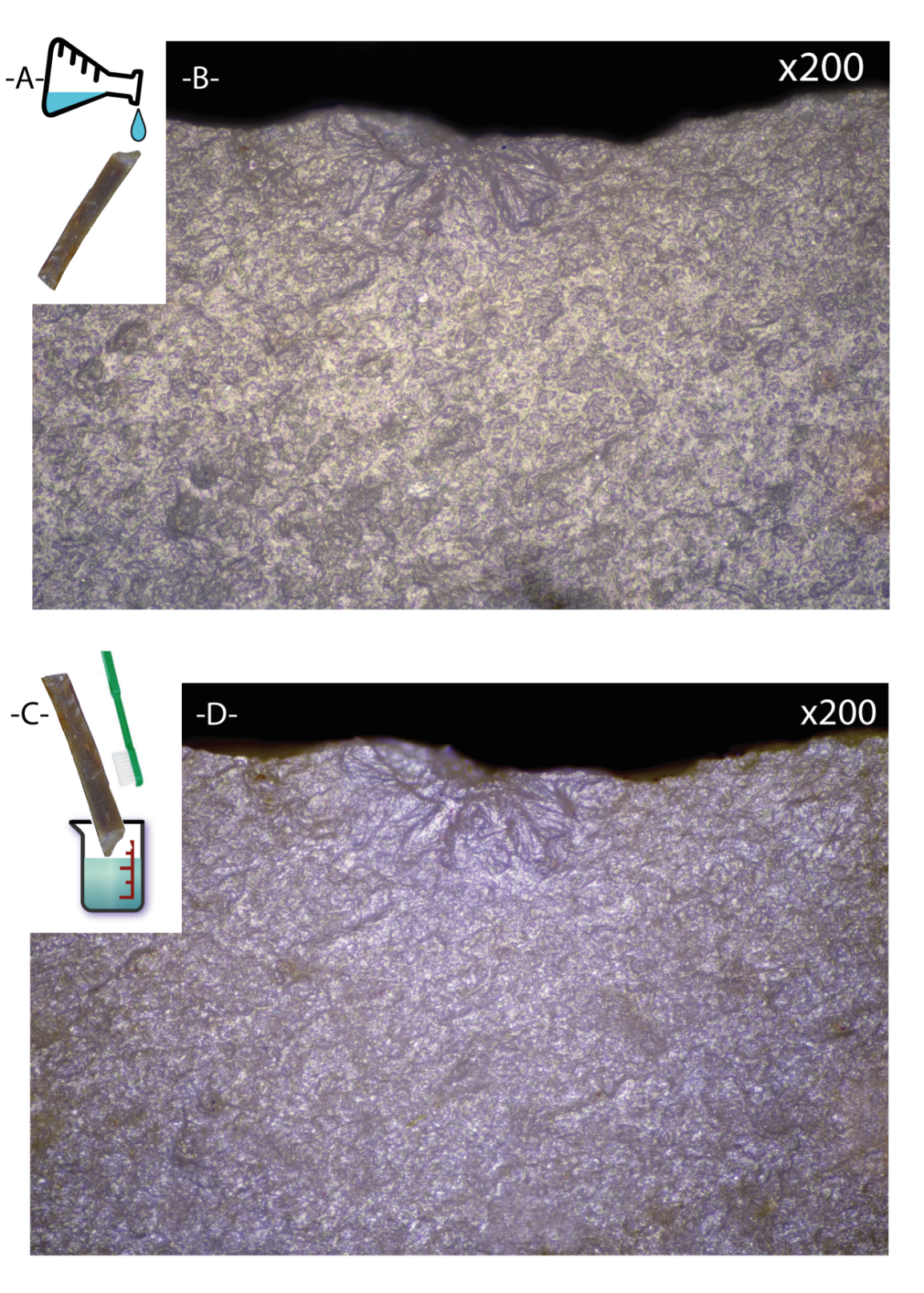


Fig. S5. Illustration of the effect of different cleaning procedures on one archaeological flint artefact from the Chatelperronian layer at Les Cottés cave in France (Soressi et al., monograph in progress). -A-: mild cleaning with pure alcohol, without using toothbrush. -B: microphotograph (magn. 200x, size: 125.05 x 81.87 mm) of the surface state after mild cleaning, showing a microscopic layer which mimics a post-depositional polish. -C-: harsh cleaning with immersion 5 minutes in a 1/10 diluted basic solution (Na₂CO₃) and with using a soft toothbrush during few seconds while rinsing with water and alcohol. -D-: microphotograph (magn. 200x, size: 141.22 x 94.23 mm) of the same spot previously photographed in -B-, after harsh cleaning procedure, revealing a well preserved surface, but affected by a slight post-depositional polish.


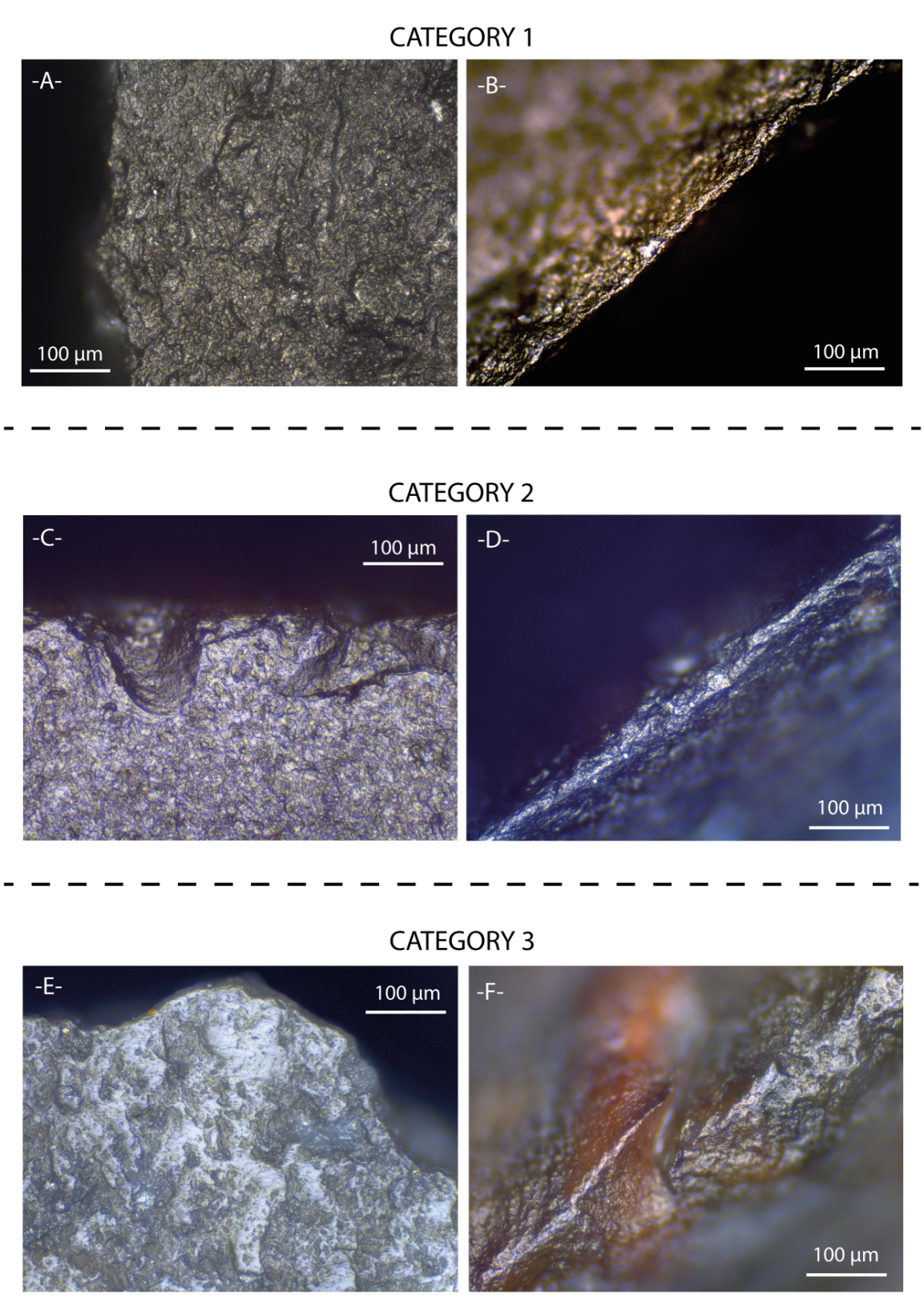


Fig. S6. Les Cottés cave, example of taphonomic classification of the sample according to three different stages of micro-surface alterations. Left: dorsal face close to the edge; right: dorsal ridge. A&B: unretouched flake US04inf (Protoaurignacian). C&D: unretouched bladelet US04inf. E&F: carinated endscraper US02 (Aurignacian). Micrographs captured by M.B. at magn. 200x under a metallurgical microscope. (N.B.: preparation of the sample involved harsh cleaning method.). Images size: 86,5 mm x 64,8 mm.
